# Supplementary material for: Protein mimetic amyloid inhibitor potently abrogates cancer-associated mutant p53 aggregation and restores tumor suppressor function
Source: Nat Commun. 2021 Jun 25;12:3962. doi: 10.1038/s41467-021-23985-1 (PMC8233319; doi:10.1038/s41467-021-23985-1)
Supplement: Supplementary file 1 — Supplementary Information [file 41467_2021_23985_MOESM1_ESM.pdf]

SUPPLEMENTARY INFORMATION

Protein mimetic amyloid inhibitor potently abrogates cancer-associated mutant p53 aggregation and restores tumor suppressor function

L. Palanikumar[1], Laura Karpauskaite[1], Mohamed Al-Sayegh[1], Ibrahim Chehade[1], Maheen Alam[2], Sarah Hassan[1], Debabrata Maity[3], Liaqat Ali[4], Mona Kalmouni[1], Yamanappa Hunashal[5,6], Jemil Ahmed[7], Tatiana Houhou[1], Shake Karapetyan[8], Zackary Falls[9], Ram Samudrala[9], Renu Pasricha[4], Gennaro Esposito[5,10], Ahmed J. Afzal[1], Andrew D. Hamilton[3*], Sunil Kumar[7*] and Mazin Magzoub[1*]

[1]Biology Program, Division of Science, New York University Abu Dhabi, P.O. Box 129188, Saadiyat Island Campus, Abu Dhabi, United Arab Emirates

[2]Department of Biology, SBA School of Science and Engineering, Lahore University of Management Sciences, Lahore, Pakistan

[3]Department of Chemistry, New York University, New York, NY 10003, United States

[4]Core Technology Platforms, New York University Abu Dhabi, P.O. Box 129188, Saadiyat Island Campus, Abu Dhabi, United Arab Emirates

[5]Chemistry Program, Division of Science, New York University Abu Dhabi, P.O. Box 129188, Saadiyat Island Campus, Abu Dhabi, United Arab Emirates

[6]DAME, Università di Udine, P.le Kolbe, 4, 33100 Udine, Italy

[7]Department of Chemistry and Biochemistry and Knoebel Institute for Healthy Aging, The University of Denver, Denver, CO 80210, United States

[8]Physics Program, Division of Science, New York University Abu Dhabi, P.O. Box 129188, Saadiyat Island Campus, Abu Dhabi, United Arab Emirates

[9]Department of Biomedical Informatics, School of Medicine and Biomedical Sciences, State University of New York (SUNY), Buffalo, NY 14203, USA

[10]INBB, Viale Medaglie d'Oro, 305, 00136 Rome, Italy

Short Title: Protein mimetic inhibitor of mutant p53 aggregation

Key Words: α-helix mimetics, amyloid, apoptosis, cancer therapeutics, cell cycle arrest, DNA-binding domain, oligopyridylamides, mutant p53, pancreatic cancer, protein aggregation, tumor suppressor

*Correspondence: mazin.magzoub@nyu.edu, sunil.kumar97@du.edu, andrew.hamilton@nyu.edu

TABLE OF CONTENTS

SECTION 1. Supplementary amyloid aggregation data

Supplementary Figure 1. Effects of oligopyridylamides on amyloid formation of aggregation prone region of p53 DBD

SECTION 2. Supplementary NMR data

Supplementary Figure 2. ^{15}N-^{1}H HSQC spectrum of 53 mM WT p53 DBD in 50 mM Tris, 200 mM KCl, 5 mM DTT (pH 7.5), recorded at 14 T (600 MHz ^{1}H frequency) and 293.2 K

Supplementary Figure 3. ^{15}N-^{1}H HSQC spectra of 45 μM WT and 41 μM mutant R248W p53 DBDs in 47 mM phosphate buffer, 141 mM KCl, 4.7 mM DTT (pH 6.8), recorded at 14 T (600 MHz ^{1}H frequency) and 293.2 K

Supplementary Figure 4. ^{15}N-^{1}H HSQC spectra of 19 μM WT and 24 μM R248W p53 DBDs in H_2O/D_2O (96/4), 16.7 mM DTT, pH 5.6 (WT DBD) or 5.8 (mutant DBD), recorded at 14 T (600 MHz ^{1}H frequency) and 293.2 K

Supplementary Figure 5. Chemical Shift Perturbation (CSP) values from ^{15}N-^{1}H HSQC spectra of WT and mutant p53 DBDs in the presence of ADH-6 at the indicated protein-to-ligand ratios

Supplementary Figure 6. ^{15}N-^{1}H HSQC maps of 18.5 μM WT and 23.5 μM R248W p53 DBDs in H_2O/D_2O (96/4), 16.4 mM DTT, pH 5.6 (WT DBD) or 5.8 (mutant DBD), following the addition of aqueous ADH-6 reaching a protein-to-ligand ratio of 1:30

SECTION 3. Supplementary intracellular aggregation, target engagement and cell viability/toxicity analysis

Supplementary Figure 7. Effects of ADH-1 and ReACp53 on cytosolic mutant p53 aggregates in MIA PaCa-2 cells

Supplementary Figure 8. ADH-6 reduces puncta in plant cells expressing mutant, but not WT, p53 DBD

Supplementary Figure 9. Western blot analysis of effects of ADH-6 on intracellular mutant R248W p53 aggregation

Supplementary Figure 10. Cellular thermal shift assay (CETSA) analysis of target engagement in MIA PaCa-2 and SK-BR-3 cells

Supplementary Figure 11. Effects of the oligopyridylamides on cancer cells harboring WT or mutant (R248W) p53

Supplementary Figure 12. Effects of aggregation-prone mutant p53 transfection on cancer cell susceptibility to ADH-6-mediated cytotoxicity

Supplementary Figure 13. Gating strategies for cell sorting

SECTION 4. Supplementary transcriptome and proteome analysis

Supplementary Table 1. Complete list of primers used

Supplementary Figure 14. ChIP-qPCR analysis of recruitment of mutant R248W p53 to the WT protein's binding sites on promoters/enhancers of target genes

Supplementary Figure 15. Western blot analysis of expression of direct p53 targets in oligopyridylamide-treated MIA PaCa-2 cells

Supplementary Figure 16. Determination of the best condition for differential gene expression analysis

Supplementary Figure 17. Identification of transcriptional regulators of dysregulated genes in oligopyridylamide-treated MIA PaCa-2 cells

Supplementary Figure 18. Phosphoproteome analysis of ADH-6 treated MIA PaCa-2 cells

Supplementary Figure 19. A simplified model of p53-mediated regulation of DNA replication/ repair and cell cycle progression/proliferation

Supplementary Table 2. Biological roles of downregulated/upregulated phosphoproteins in DNA repair/replication and cycle progression/proliferation

SECTION 5. Supplementary *in vivo* tumor reduction data

Supplementary Figure 20. Effects of ADH-6 on MIA PaCa-2 xenografts *in vivo*
Supplementary Figure 21. Effects of ADH-6 on SK-BR-3 xenografts *in vivo*
Supplementary Figure 22. Histological analysis of vital organs following treatment with lower doses of ADH-6
Supplementary Figure 23. Histological analysis of vital organs following treatment with higher doses of ADH-6

SECTION 6. Synthesis and characterization of ADH-6

Supplementary Figure 24. ^{1}H NMR of ADH-6-NCS
Supplementary Figure 25. ^{1}H NMR of ADH-6_F (ADH-6_{FITC})

SECTION 1. Supplementary amyloid aggregation data

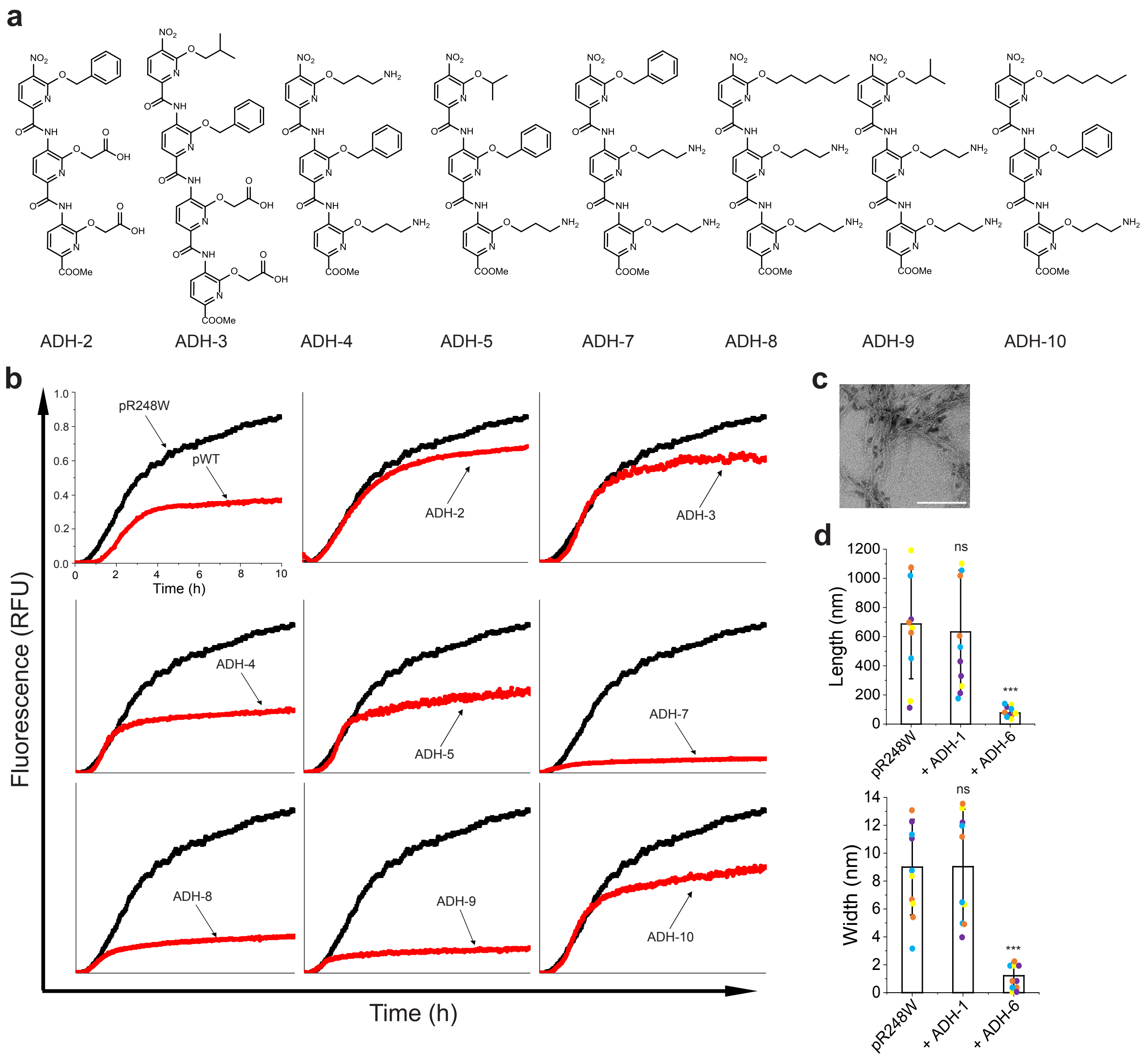

Supplementary Figure 1. Effects of oligopyridylamides on amyloid formation of aggregation-prone region of p53 DBD. (a) Chemical structures of the oligopyridylamides used in this study (except ADH-1 and ADH-6, which are shown in Figure 1d). (**b**) Effects of the oligopyridylamides on pR248W amyloid formation. Kinetic profiles for aggregation of 25 µM pR248W vs pWT, and for 25 µM pR248W in the absence or presence of an equimolar amount of the oligopyridylamides co-mixed at the start of the reaction. Aggregation profiles were acquired by measuring the fluorescence of the thioflavin T (ThT) reporter ($\lambda_{ex/em}$ = 440/480 nm) at 5-min intervals at 37 °C (n = 4). (**c**) Representative transmission electron microscopy (TEM) image for aggregation of 25 µM pR248W in the presence of an equimolar amount of ADH-6 added during the growth phase of (i.e. 5 h after the start of the reaction). TEM image was acquired at 10 h after the start of the aggregation reaction. Scale bar = 500 nm. (**d**). Quantification of fibril lengths and widths for 25 µM pR248W in the absence or presence of an equimolar amount of ADH-1 or ADH-6 added during the growth phase (i.e. 5 h after the start of the reaction; representative TEM images shown in Figure 1f). Data presented are mean ± SD (n = 10 biologically independent samples). Statistical analysis was performed using one-way ANOVA followed by Dunnett's *post hoc* test. P = 0.0007 for ADH-6 vs pR248W fibril length comparison, and P < 0.0001 for ADH-6 vs pR248W fibril width comparison. ***P < 0.001 or non-significant (ns, P > 0.05) compared with pR248W controls.

SECTION 2. Supplementary NMR data

The initial ^{15}N-^{1}H HSQC NMR spectra of p53 DBD obtained in Tris buffer showed several differences compared to the maps reported by Rasquinha *et al.*[1] Besides numerous chemical shift displacements, the resonance intensities appeared weak and smeared despite the sample concentration not being particularly low (50 μM) (Supplementary Figure 2). As was previously reported, studying the NMR spectrum of p53 DBD is challenging due to the inherent instability of the domain[1,2], which is responsible for the conformational heterogeneity over intermediate timescales that leads to substantial line broadening of some signals.

In order to reproduce the spectral quality previously obtained[1], the protein sample was dialyzed to replace the Tris buffer with phosphate buffer. The quality of the HSQC contour plots improved, as did the comparability with the previously published spectra (Supplementary Figure 3). Most of the backbone amide assignments illustrated by Rasquinha et al.[1] could be transferred to our data. This also allowed us to recognize the presence of several new peaks, mostly clustered in the region of unstructured peptides, arising from a partially unfolded species. The onset of this species was ascribed to the effect of ultracentrifugation and buffer exchange manipulations on a protein of limited stability. Since the NMR pattern of the folded form was observed to be stable for a reasonable length of time (at least 15 days) for both the WT and R248W mutant species, notwithstanding the presence of a partially unfolded conformer, we decided to proceed to the titration with the designed oligopyridylamide ligand, namely ADH-6. However, we could not observe any effect on either protein variant due to the systematic precipitation of the oligopyridylamide after its addition to the protein samples. Regardless of the concentration of the aqueous titrant we employed to reach protein-to-ligand ratios between 1:2 and 1:10, no effect could be detected in the HSQC spectra, but the sedimentation at the bottom of the NMR tube of a white powder that had to be the added compound given the constancy of the protein spectra. The same result was obtained after adding a concentrated ADH-6 solution in DMSO to the R248W p53 DBD mutant, to reach a protein-to-ligand nominal ratio of the order of 1:100. It is worth noting that the protein did not exhibit any relevant instability effect due to 1 μL of DMSO even after 8 days.

To overcome the incompatibility of ADH-6 with p53 DBD in saline, the protein samples were further dialyzed against pure water containing only 5 mM DTT (necessary to preserve the reduced state of the 7 free Cys residues in the p53 DBD domain). Although the pH was lowered by 1 unit (for R248W p53 DBD) or 1.2 units (for WT p53 DBD) with respect to the initial value (6.8), the spectra of both proteins still showed good similarity to the literature[1]. Additionally, the partially unfolded conformers formed after the first buffer exchange were preserved (Supplementary Figure 4). The assignment by analogy could be performed and both protein samples could be challenged with an addition of aqueous ADH-6 leading to protein-to-ligand ratios of 1:11 (for WT p53 DBD) and 1:8 to 1:15 (for R248W p53 DBD). This time several specific peak shifts were observed with both proteins as illustrated in the panels of Figure 2 in the main text.

The experimental chemical shift perturbation (CSP) values that were determined after addition of ADH-6 affect, to different extents, approximately the same group of residues in the two studied variants of p53 DBD (Supplementary Figure 5). However, the interaction with ADH-6 involved also the partially unfolded species that were present in the samples of both variants (Figure 2). Due to this additional involvement and the signal-to-noise ratios, no quantitative estimate was reliably feasible to assess a binding constant of ADH-6 to the WT and mutant R248W DBDs. Finally, reaching a ligand-to-protein ratio of ~30 resulted in unfolding of both WT and mutant proteins (Supplementary Figure 6).

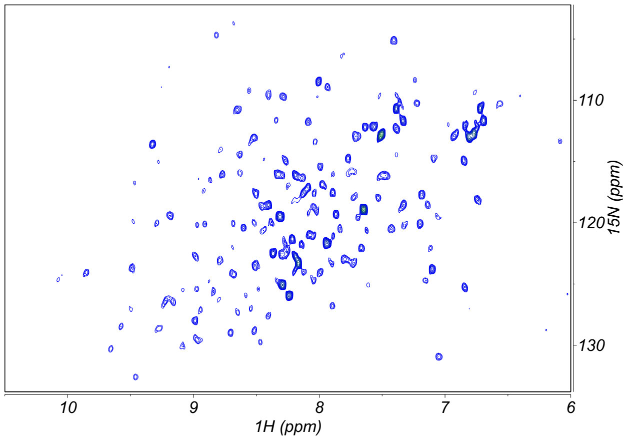

Supplementary Figure 2. ^{15}N-^{1}H HSQC spectrum of 53 µM WT p53 DBD in 50 mM Tris, 200 mM KCl, 5 mM DTT (pH 7.5), recorded at 14 T (600 MHz ^{1}H frequency) and 293.2 K.

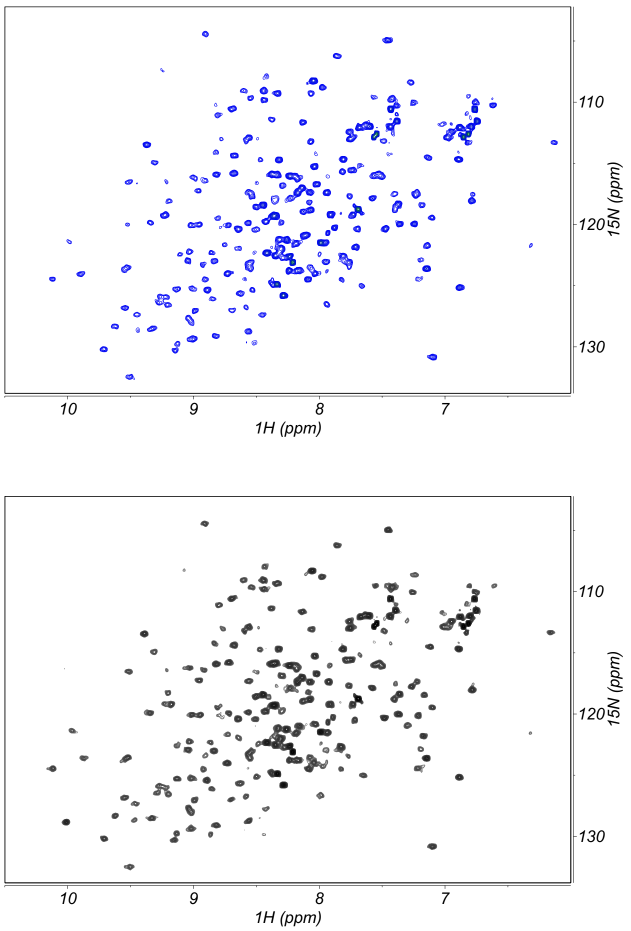

Supplementary Figure 3. ^{15}N-^{1}H HSQC spectra of 45 μM WT (*upper panel*, blue contours) and 41 μM mutant R248W (*lower panel*, black contours) p53 DBDs in 47 mM phosphate buffer, 141 mM KCl, 4.7 mM DTT (pH 6.8), recorded at 14 T (600 MHz ^{1}H frequency) and 293.2 K.

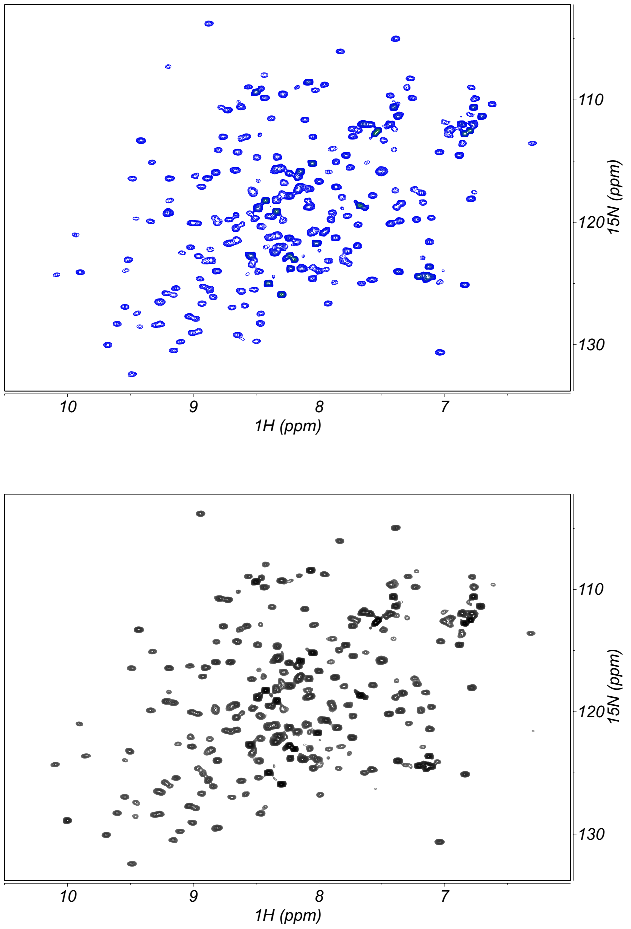

Supplementary Figure 4. ^{15}N-^{1}H HSQC spectra of 19 μM WT (*upper panel*, blue contours) and 24 μM R248W (*lower panel*, black contours) p53 DBDs in H_2O/D_2O (96/4), 16.7 mM DTT, pH 5.6 (WT DBD) or 5.8 (mutant DBD), recorded at 14 T (600 MHz ^{1}H frequency) and 293.2 K.

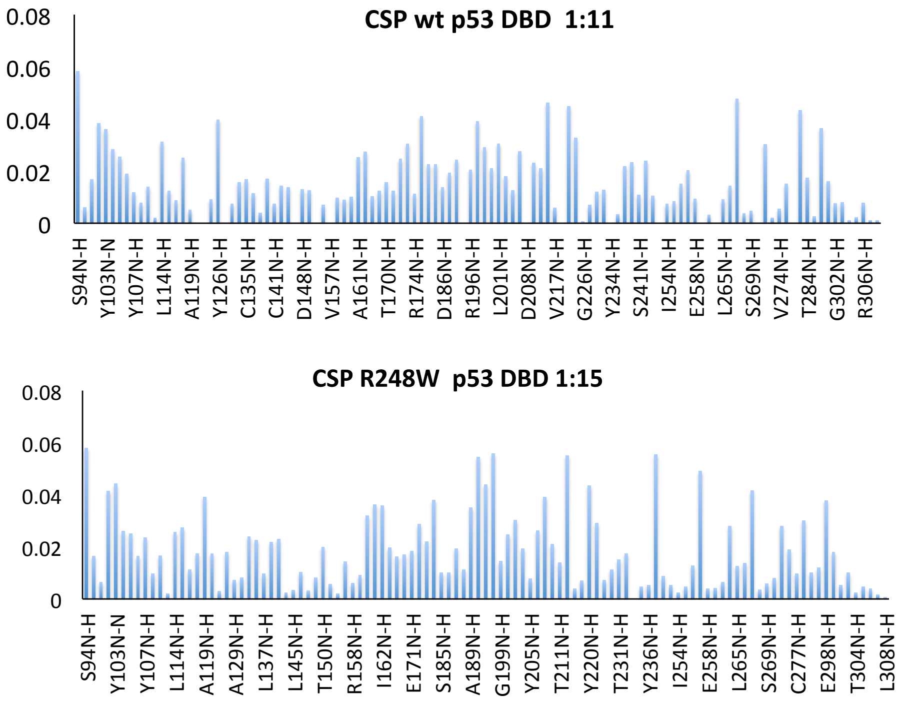

Supplementary Figure 5. Chemical Shift Perturbation (CSP) values from ^{15}N-^{1}H HSQC spectra of WT and R248W mutant p53 DBDs in the presence of ADH-6 at the indicated protein-to-ligand ratios.

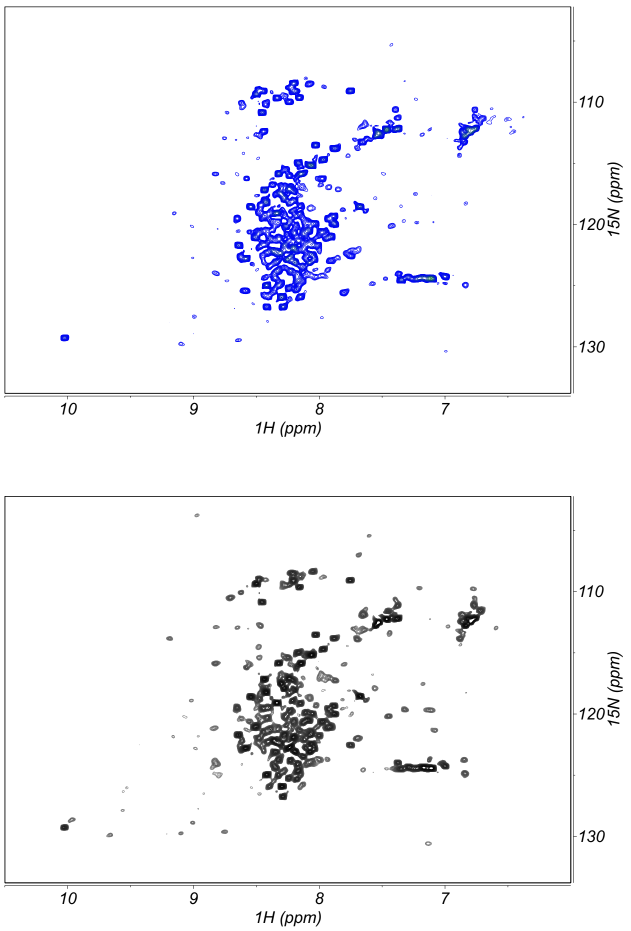

Supplementary Figure 6. ^{15}N-^{1}H HSQC maps of 18.5 μM WT (*upper panel*, blue contours) and 23.5 μM R248W (*lower panel*, black contours) p53 DBDs in H_2O/D_2O (96/4), 16.4 mM DTT, pH 5.6 (WT DBD) or 5.8 (mutant DBD), following the addition of aqueous ADH-6 reaching a protein-to-ligand ratio of 1:30. Both proteins appear extensively unfolded. The spectra were recorded at 14 T (600 MHz ^{1}H frequency) and 293.2 K.

SECTION 3. Supplementary intracellular aggregation, target engagement and cell viability/toxicity analysis

3.1. Supplementary intracellular mutant p53 aggregation analysis

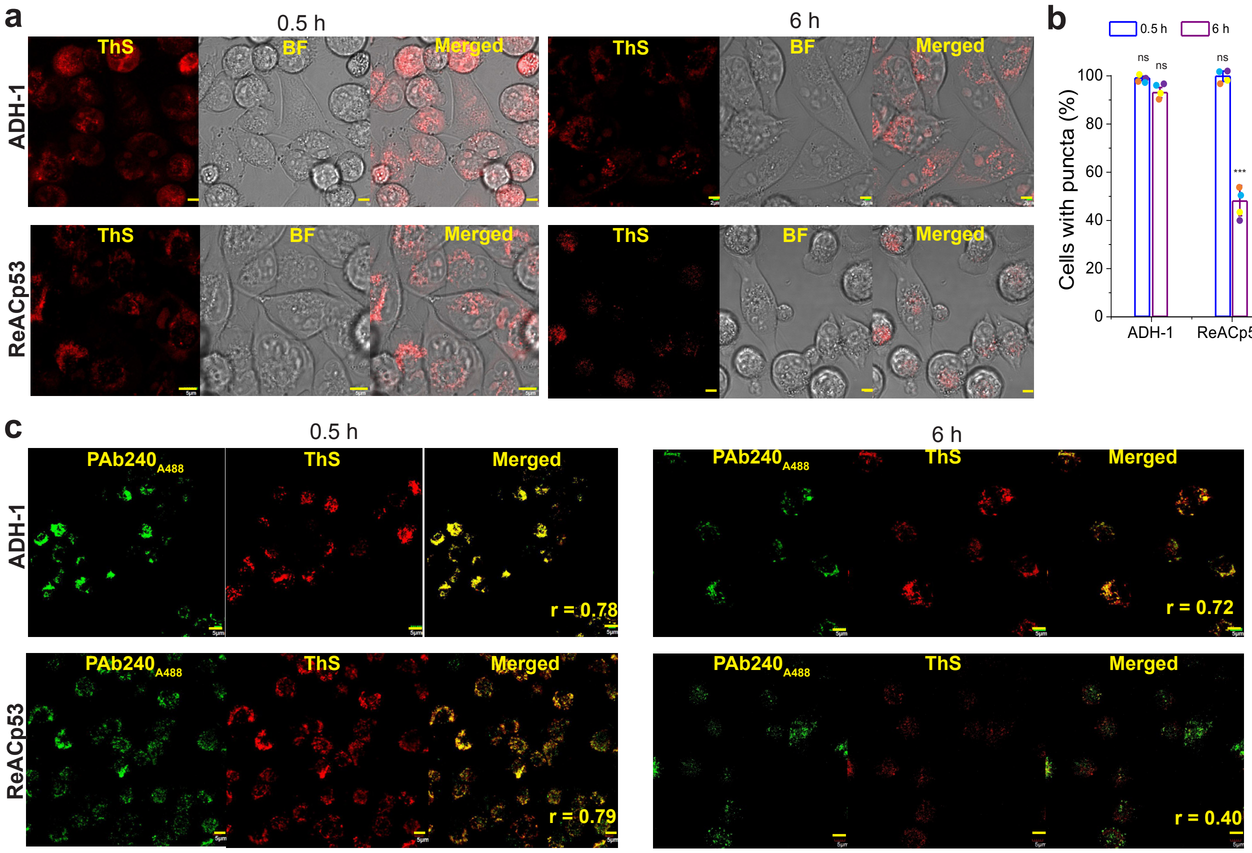

Supplementary Figure 7. Effects of ADH-1 and ReACp53 on cytosolic mutant p53 aggregates in mutant p53 bearing cancer cells. (**a**) Confocal fluorescence microscopy images showing thioflavin S (ThS) staining of mutant p53 (R248W) aggregates in MIA PaCa-2 cells treated with vehicle (0.02% DMSO) or 5 µM ADH-1 or ReACp53 for the indicated durations. Imaging experiments were performed in triplicate and representative images are shown. **(b)** Quantification of ThS-positive MIA PaCa-2 cells after treatment with ADH-1 or ReACp53. The number of positively stained cells in 3–5 different fields of view are expressed as % of the total number of cells (n = 4 biologically independent samples). Data presented are mean ± SD. Statistical analysis was performed using one-way ANOVA followed by Dunnett's *post hoc* test. $P < 0.0001$ for ReACp53 vs vehicle at 6 h. (**c**) Confocal fluorescence microscopy images of ThS and PAb 240 antibody staining of R248W aggregates in MIA PaCa-2, treated with 5 µM ADH-1, ReACp53 or ADH-6 for the 0.5 or 6 h. Images shown are representative of four independent experiments. Colocalization was quantified using directional Pearson correlation coefficient, r (ref 3). Scale bar = 5 µM. ***$P < 0.001$ or non-significant (ns, $P > 0.05$) for comparisons with vehicle-treated controls.

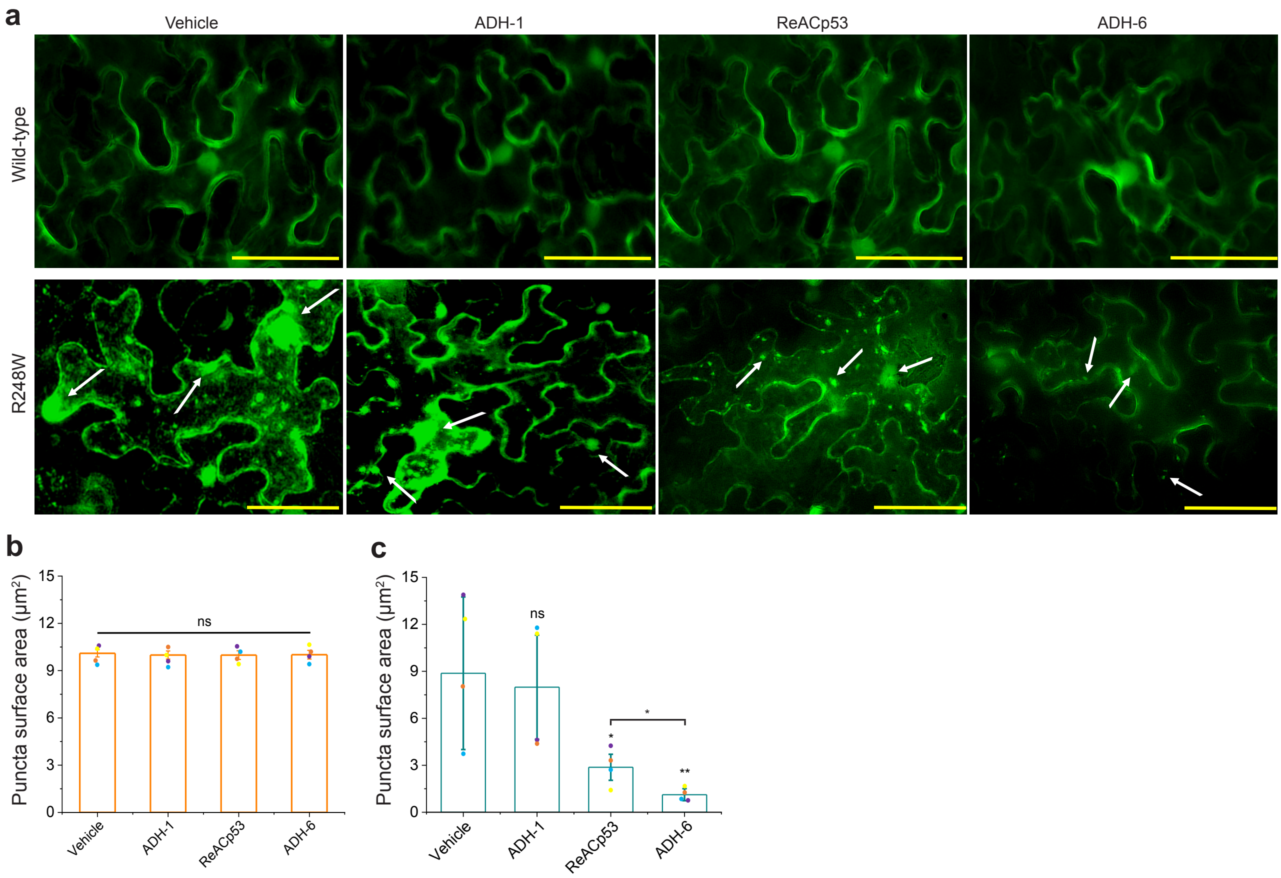

Supplementary Figure 8. ADH-6 reduces puncta in plant cells expressing mutant, but not WT, p53 DBD. YFP:p53DBD^{wt} and YFP:p53DBD^{R248W} (YFP-tagged WT and mutant R248W p53 DBDs, respectively) were expressed from the 35S-promoter by agroinfiltrations in *Nicotiana benthamiana* leaves. YFP:p53DBD^{WT} and YFP:p53DBD^{R248W} were infiltrated at an OD of 1.0. 5 µM ADH-1, ReACp53 and ADH-6 were introduced 48 hpi into the bacterial infiltrated region of the leaf, and the effects of the treatments on YFP:p53DBD^{WT} and YFP:p53DBD^{R248W} puncta were observed 24 h later using confocal fluorescence microscopy. (**a**) Images of *N. benthamiana* leaves expressing YFP:p53DBD^{WT} (*upper panels*) or YFP:p53DBD^{R248W} (*lower panels*) treated with ADH-1, ReACp53 or ADH-6. Images shown are representative of four independent experiments. Scale bar = 50 µm. (**b**,**c**) Effects of the treatments on YFP:p53DBD^{WT} (**b**) and YFP:p53DBD^{R248W} (**c**) puncta in *N. benthamiana* leaves. Puncta sizes were quantified in 3–5 different fields of view ($n = 4$). Data presented are mean ± SD. Statistical analysis was performed using one-way ANOVA followed by Tukey's *post hoc* test. $P = 0.0077$ for ADH-6 vs vehicle, $P = 0.0333$ for ReACp53 vs vehicle, and $P = 0.0328$ for ADH-6 vs ReACp53. *$P < 0.05$, **$P < 0.01$ or non-significant (ns, $P > 0.05$) for comparisons with vehicle-treated controls and amongst treatment groups.

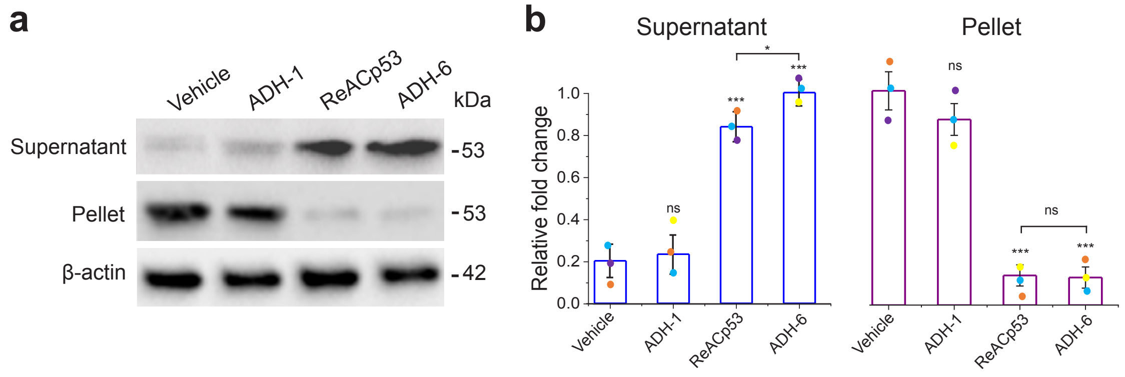

Supplementary Figure 9. Western blot analysis of effects of ADH-6 on intracellular mutant R248W p53 aggregation. (**a**) Immunoblots of mutant R248W p53 in the soluble (supernatant) and insoluble (pellet) fractions of MIA PaCa-2 cells treated with vehicle or 5 µM ADH-1, ReACp53 or ADH-6 for 6 h and detected by the anti-p53 antibody DO-7. Immunoblots shown are representative of three independent experiments. (**b**) Densitometric quantification of the immunoblot bands of mutant p53 in the soluble (*left panel*) and insoluble (*right panel*) fractions (n = 3). Data presented are mean ± SD. Statistical analysis was performed using one-way ANOVA followed by Tukey's *post hoc* test. $P < 0.0001$ for ADH-6 vs vehicle and ReACp53 vs vehicle, and $P = 0.0389$ for ADH-6 vs ReACp53 (supernatant); $P < 0.0001$ for ADH-6 vs vehicle and ReACp53 vs vehicle (pellet). *P < 0.05, ***P < 0.001 or non-significant (ns, $P > 0.05$) for comparisons with vehicle-treated controls and amongst treatment groups.

3.2. Supplementary intracellular target engagement analysis

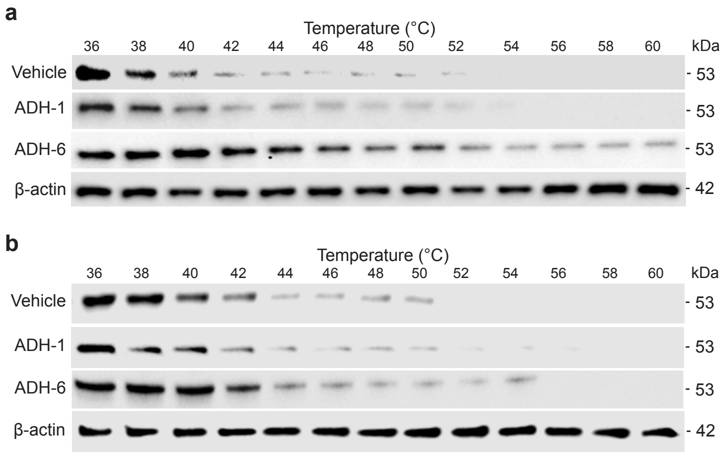

Supplementary Figure 10. Cellular thermal shift assay (CETSA) analysis of target engagement in MIA PaCa-2 and SK-BR-3 cells. (**a**) Immunoblots of mutant R248W (**a**) and R175H (**b**) p53 in the soluble fractions of MIA PaCa-2 and SK-BR-3 cells, respectively, following treatment with vehicle or 5 µM ADH-1 or ADH-6 for 6 h and then heating to the indicated temperatures for 3 min. Mutant p53 was detected by the anti-p53 antibody DO-7. Immunoblots shown in (**a**) and (**b**) are representative of three independent experiments. Densitometric quantification of the immunoblot bands was used to generate the melting curves shown in Figure 3h,i.

3.3. Supplementary cell viability/toxicity data

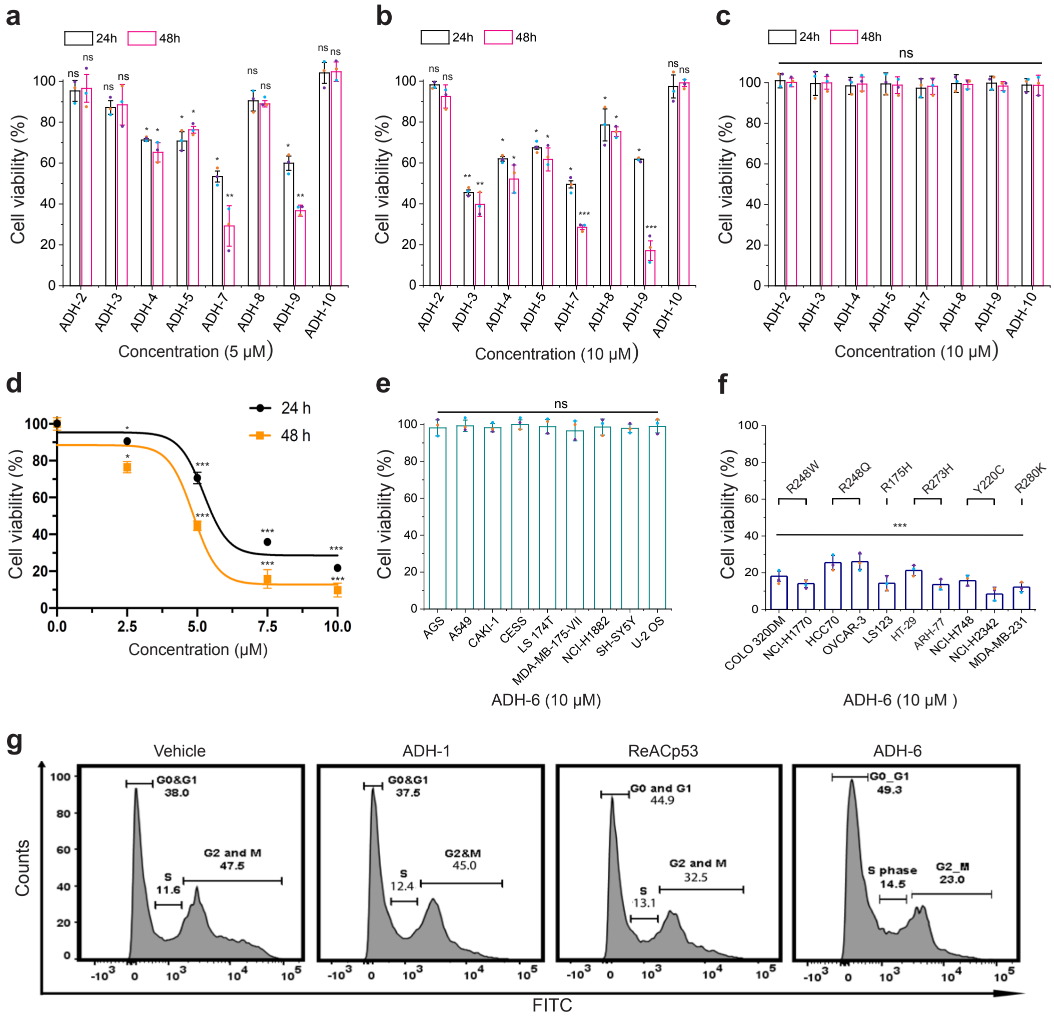

Supplementary Figure 11. Effects of the oligopyridylamides on cancer cells harboring WT or mutant (R248W) p53. (**a**–**c**) Screen to identify oligopyridylamides that are toxic to cancer cells bearing mutant, but not WT, p53. MIA PaCa-2 (mutant R248W p53) (**a**,**b**) or MCF-7 (WT p53) (**c**) cells were treated with the indicated concentrations of ADH compounds for 24 or 48 h. Cell viability was assessed using the MTS assay (n = 3). Data presented are mean ± SD. Statistical analysis was performed using one-way ANOVA followed by Dunnett's *post hoc* test. **P* < 0.05, ***P* < 0.01, ****P* < 0.001 or non-significant (ns, P > 0.05) compared with controls. (**d**) Effects of control peptide ReACp53 on cancer cells bearing mutant p53. MIA PaCa-2 cells were treated with the indicated concentrations of ReAcp53 for 24 or 48 h. Cell viability was assessed using the MTS assay (mean ± SD; n = 3). Two-tailed unpaired t-test: **P* < 0.05 or ****P* < 0.001 for comparisons with ADH-1 treatment of MIA PaCa-2 cells at the same concentration and incubation time (ADH-1 data shown in Figure 4a). (**e**,**f**) Probing the effects of ADH-6 on viability of different cancer cells bearing WT or mutant p53. Bone (U-2 OS), brain (SH-SY5Y), breast (MDA-MB-175-VII), colon (LS 174T), gastric (AGS), renal (CAKI-1), leukemia (CESS) and lung (A549 and NCI-H1882), cancer cells harboring WT p53 (**e**), or aggregation-prone R248W (colon: COLO 320DM; lung: NCI-H1770), R248Q (breast: HCC70; ovarian: OVCAR-3), R175H (colon: LS123), R273H (colon: HT-29; leukemia: ARH-77), Y220C (lung: NCI-H748 and NCI-H2342) and R280K (breast: MDA-MB-231), mutant p53 harboring cancer cells (**f**), were treated with 10 μM ADH-6 for 48 h. Cell viability was assessed using the MTS assay (mean ± SD; n = 3). One-way ANOVA followed by Dunnett's *post hoc* test: ****P* < 0.001 or non-significant (ns, P > 0.05) compared with controls. (**g**) Effects of ADH-6 on cell cycle distribution of mutant p53 bearing cancer cells. MIA PaCa-2 cells were treated with vehicle or 5 μM ADH-1, ReACp53 or ADH-6 for 6 h. Cell cycle distribution of the cells was then evaluated using a cell cycle assay kit (Abcam), with measurements done on a BD FACSAria III cell sorter (n = 4). Shown are representative flow cytometry histograms for the different treatment groups.

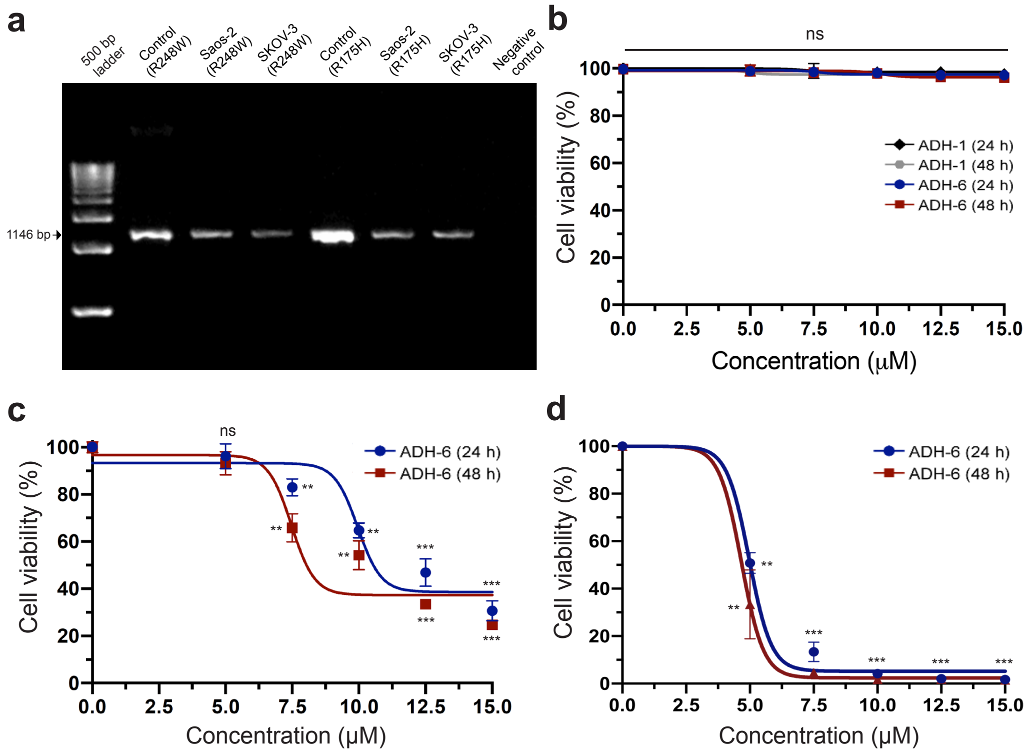

Supplementary Figure 12. Effects of aggregation-prone mutant p53 transfection on cancer cell susceptibility to ADH-6-mediated cytotoxicity. (**a**) Verification of successful transfection of p53 null Saos-2 and SKOV-3 cells with mutant R248W or R175H p53. The vectors were purified from the transfected cells, and the amplified *amp*^{r} PCR product was electrophoresed on a 3% agarose gel containing GelGreen Nucleic Acid Stain. Gel shown is representative of three independent experiments. (**b**–**d**) Viability of SKOV-3 cells before (**b**) and after transfection with mutant R248W (**c**) or R175H (**d**) p53 treated with increasing concentrations of ADH-6 for 24 or 48 h. Cell viability in (**b**–**d**) was assessed using the MTS assay, with the % viability determined form the ratio of the absorbance of the treated cells to the control cells (n = 3 biologically independent samples). Data presented are mean ± SD. Statistical analysis was performed using two-tailed unpaired t-test. **P < 0.01, ***P < 0.001 or non-significant (ns, P > 0.05) for ADH-6 treatment of SKOV-3/R248W (**c**) or SKOV-3/R175H (**d**) compared with untransfected cells (**b**) at the same compound concentration (2.5–10 μM) and incubation time (24 or 48 h).

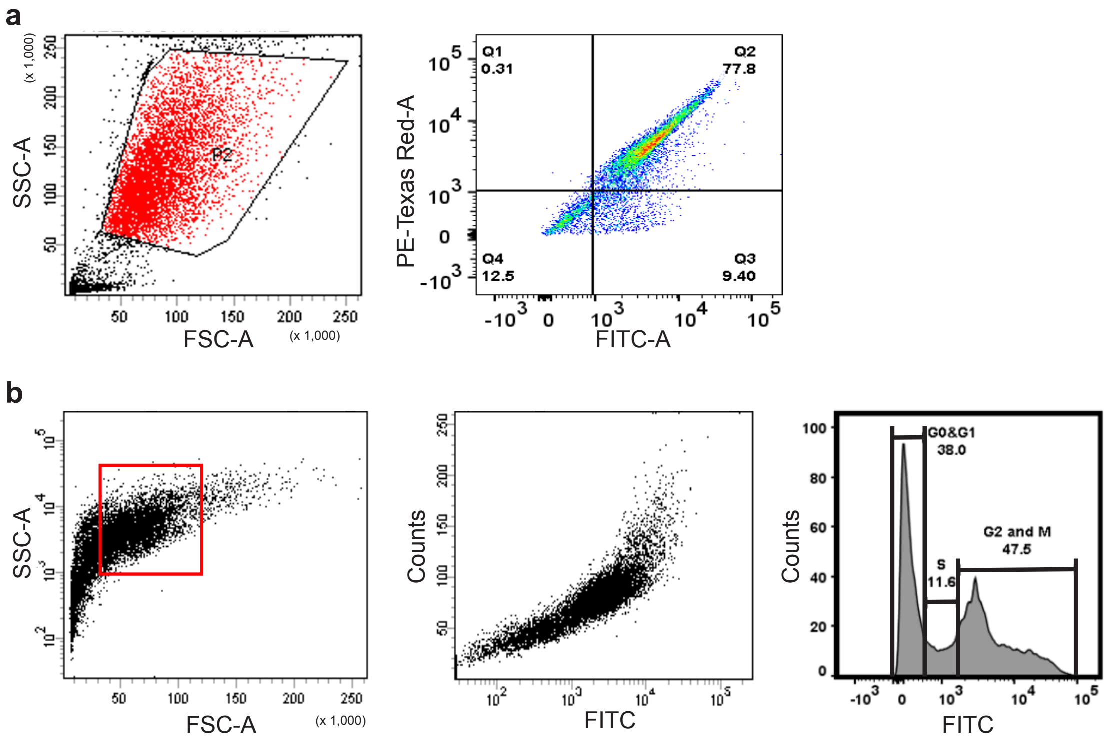

Supplementary Figure 13. Gating strategies for cell sorting. Gating strategies for eliminating cell debris from the analysis, followed by detecting live, apoptotic and necrotic cells using annexin V/propidium iodide (PI) staining (**a**) or determining cell cycle distribution using Nuclear Green CCS1 staining (**b**). The gating strategy in (**a**) was used for the apoptosis assay presented in Figure 4g,h, and the strategy in (**b**) was used for the cell cycle analysis presented in Figure 4i and Supplementary Figure 11g.

SECTION 4. Supplementary transactivation analysis

4.1. Chromatin immunoprecipitation (ChIP) with quantitative real-time PCR (ChIP-qPCR) assay

ChIP-qPCR (primers used are listed in Supplementary Table 1) revealed that treatment of MIA PaCa-2 cells with ADH-6 resulted in binding of mutant R248W p53 to the WT protein's binding sites on promoters/enhancers of target genes, *CDKN1A* (also known as *P21*), *PIG3* and *NOXA* (Supplementary Figure 14). p21, a cyclin-dependent kinase inhibitor, is required for p53-mediated cell cycle arrest in response to DNA damage and other cellular stresses[4,5]. *PIG3* (p53-induced gene 3) is involved in both the early cellular response to DNA damage and p53-induced apoptosis[6,7]. *NOXA* (Latin for damage) encodes a member of the Bcl-2 family of apoptosis regulator proteins; upon p53-induced expression, Noxa localizes to mitochondria, where the protein binds to and inhibits anti-apoptotic Bcl-2 family members[8]. Notably, Noxa has also been identified as a key determinant of cytotoxic response of cancer cells to standard chemotherapy and targeted cancer therapy[9,10].

The ChIP-qPCR results were confirmed using western blot, which showed elevated expression of both p21 and Noxa in ADH-6 treated MIA PaCa-2 cells (Supplementary Figure 15). Interestingly, we also observed significantly higher expression of p53-inducible MDM2 and Bax in response to ADH-6 treatment (Supplementary Figure 15). Bax is another member of the Bcl-2 family, which, once activated, induces permeabilization of the outer mitochondrial membrane, leading to release of cytochrome c and activation of the apoptosis initiator caspase-9[11]. Surprisingly, recruitment to the *MDM2* and *BAX* genes was not observed by ChIP-qPCR. This could be due to the interaction of mutant p53 with these genes being too transient or weak to be detected by the assay[12,13].

Supplementary Table 1. Complete list of primers used

Supplementary Table 1a. Primers used for the plant cell studies			
Primers for PCR amplification of *TP53* DBD	Forward		**cacc**TCATCTTCTGTCCCTTCCCAGAAAACC
	Reverse		**tca**GGGCAGCTCGTGGTGAGGCTCCCCT
M13	Forward		GTAAAACGACGGCCAG
	Reverse		CAGGAAACAGCTATGAC
**Supplementary Table 1b. Primers used for the cancer cell transfection studies**			
Primers for PCR amplification of *amp*^{r}	Forward		AGATTATCAAAAAGGATCTTCACCT
	Reverse		CCTCGTGATACGCCTATTTTTATAG
**Supplementary Table 1c. Primers used for ChIP-qPCR studies**			
**Mutant R248W p53**			
**Gene**	**Primer**	**References**	**Sequence**
CDKN1A (P21)	Forward	14	GTGGCTCTGATTGGCTTTCTG
	Reverse		CTGAAAACAGGCAGCCCAAG
BAX	Forward	15	TAATCCCAGCGCTTTGGAA
	Reverse		TGCAGAGACCTGGATCTAGCAA
PUMA	Forward	16	GCGAGACTGTGGCCTTGTGT
	Reverse		CGTTCCAGGGTCCACAAAGT
NOXA	Forward	11	CAGCGTTTGCAGATGGTCAA
	Reverse		CCCCGAAATTACTTCCTTACAAAA
PIG3	Forward	12	CACTCCCAACGCCTCCTTT
	Reverse		GCCCATCTTGAGCATGGGT
MDM2	Forward	17	GGTTGACTCAGCTTTTCCTCTTG
	Reverse		GGAAAATGCATGGTTTAAATAGCC
GADD45	Forward	18	AGCGGAAGAGATCCCTGTGA
	Reverse		CGGGAGGCAGGCAGATG
**p63**			
**Gene**	**Primer**	**References**	**Sequence**
CDKN1A (P21)	Forward	19	GTGGCTCTGATTGGCTTTCTG
	Reverse		CTGAAAACAGGCAGCCCAAG
**p73**			
**Gene**	**Primer**	**References**	**Sequence**
CDKN1A (P21)	Forward	20	GTGGCTCTGATTGGCTTTCT
	Reverse		AGCCTCTTCTATGCCAGAGC

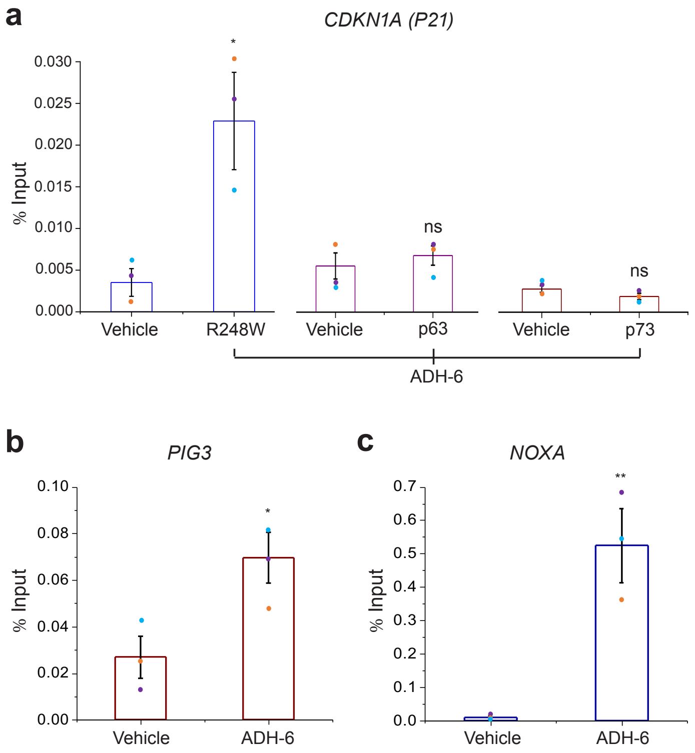

Supplementary Figure 14. ChIP-qPCR analysis of recruitment of mutant R248W p53 to the WT protein's binding sites on promoters/enhancers of target genes. Binding of mutant R248W p53 to the WT protein's transcriptional targets, *CDKN1A* (*P21*) (**a**), *PIG3* (**b**) and *NOXA* (**c**), in MIA PaCa-2 cells treated with vehicle or 5 µM ADH-6 for 24 h. As a control, binding of p63 and p73 to *CDKN1A* in ADH-6 treated MIA PaCa-2 cells is also presented (**a**). Data presented are mean ± SD; $n = 3$ biologically independent samples. Statistical analysis was performed using two-tailed unpaired *t*-test. $P = 0.0332$ for ADH-6/R248W vs vehicle (**a**), $P = 0.04$ for ADH-6 vs vehicle (**b**), and $P = 0.0093$ for ADH-6 vs vehicle (**c**). *$P < 0.05$, **$P < 0.01$ or non-significant (ns, $P > 0.05$) for comparisons with vehicle-treated controls.

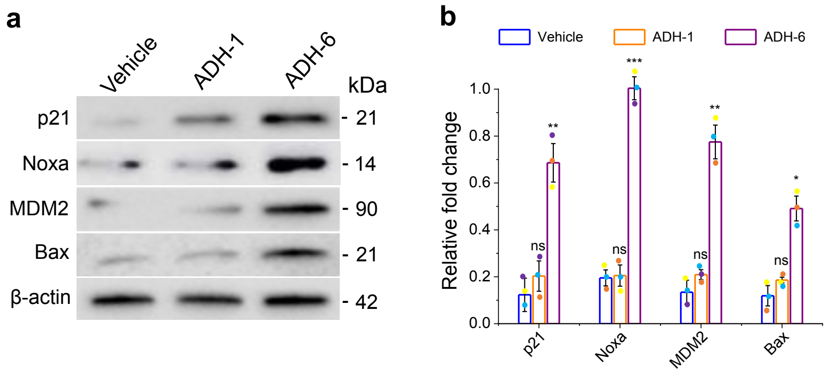

Supplementary Figure 15. Western blot analysis of expression of direct p53 targets in oligopyridylamide-treated MIA PaCa-2 cells. (**a**) Immunoblots of p21, Noxa, MDM2 and Bax in MIA PaCa-2 cells treated with vehicle or 5 µM ADH-1 or ADH-6 for 24 h. Immunoblots shown are representative of three independent experiments. (**b**) Densitometric quantification of the immunoblot bands of the p53 targets (n = 3). Data presented are mean ± SD. Statistical analysis was performed using one-way ANOVA followed by Dunnett's *post hoc* test. ADH-6 vs vehicle: P = 0.0013, 0.0002, 0.001 and 0.0403 for p21, Noxa, MDM2 and Bax, respectively. *P < 0.05, **P < 0.01, ***P < 0.001 or non-significant (ns, P > 0.05) for comparisons with controls.

4.2. Supplementary transcriptome analysis

RNA-Seq analysis showed that ADH-6 treatment of mutant R248W p53-bearing MIA PaCa-2 cells led to significant upregulation of p53 target genes, *CDKN1A*, *TP53INP1*, *FOS* and *EGR1* (Figure 5e). Upon translation, tumor protein p53-inducible nuclear protein 1 (TP53INP1) forms a protein complex with homeodomain-interacting protein kinase-2 (HIPK2) or protein kinase C δ (PKCδ) that phosphorylates p53 at residue S46[21]. This stabilizes p53 and enhances its activity, which leads to transcriptional activation of target genes, such as *CDKN1A*, and subsequent cell cycle arrest and apoptosis[21]. The first intron of the *FOS gene* contains a p53-responsive element, and overexpression of p53 has been shown to induce *FOS* upregulation, which contributes to both p53-mediated apoptosis and cell cycle arrest[22]. EGR1 is a transcriptional regulator that can be directly activated by p53 via a non-consensus p53 binding site on the *EGR1* promoter. Although EGR1 has a range of roles, its genotoxic stress-induced upregulation results in apoptosis in most mammalian cells[23].

On the other hand, ADH-6 caused significant downregulation of *TP73* and *SIX1* compared to controls (Figure 5e). Of note, downregulation of p73, whose overexpression is associated with advanced-stage cancer[24], was also observed following treatment of mutant p53-bearing cells with ReACp53[25]. SIX1 is a homeodomain-containing transcription factor that regulates cell migration, invasion and proliferation in progenitor cell populations, but is not expressed in normal adult tissue[26]. Importantly, SIX1 is re-expressed in many cancers and acts as a p53 down-regulator through an MDM2-independent pathway[26].

Other enrichments, such as isoprenoid biosynthesis (GO:0008299), lipid biosynthesis (GO:0008610) and cholesterol biosynthesis (GO:0006695) were also identified in ADH-6 treated cells (Supplementary Figure 16c,d). The heatmap generated from genes involved in these processes revealed upregulation of the mevalonate pathway, which is essential for cancer cell survival and growth[27]. Of relevance, the mevalonate pathway is mediated by non-aggregated mutant p53[28]. Thus, ADH-6-induced release of overexpressed mutant p53 from the cellular inclusions likely leads to activation of some oncogenic pathways. However, it appears that the bulk of rescued mutant p53 behaves similar to the WT protein and upregulates major tumor-suppressive pathways, which overwhelms the pro-tumor activities and results in the observed inhibition of proliferation via cell cycle arrest and induction of apoptosis (Figures 4,5 and Supplementary Figures 11,17).

a

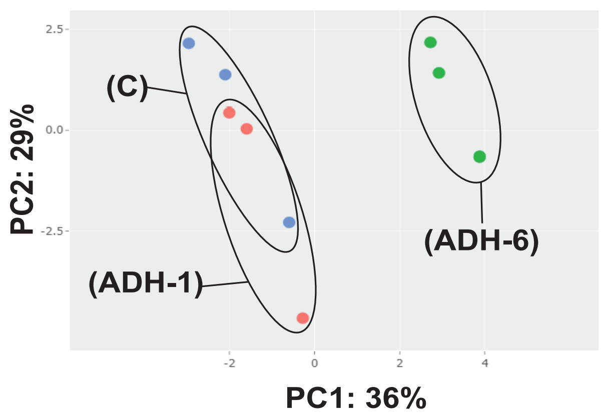

b

Conditions	*P*-adj cutoff	Number of DEGs
ADH-6/ADH-1	*P*-adj< 0.05	364
	P-adj< 0.01	172
	P-adj< 0.001	97
ADH-6/C	*P*-adj< 0.05	485
	P-adj< 0.01	274
	P-adj< 0.001	108
ADH-1/C	*P*-adj< 0.05	289
	P-adj< 0.01	51
	P-adj< 0.001	4

c

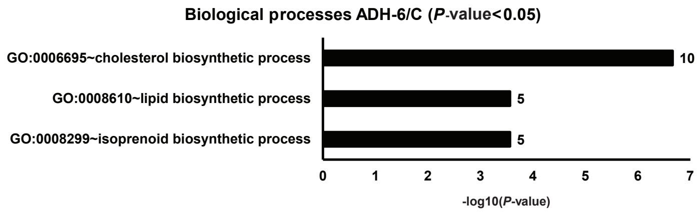

d

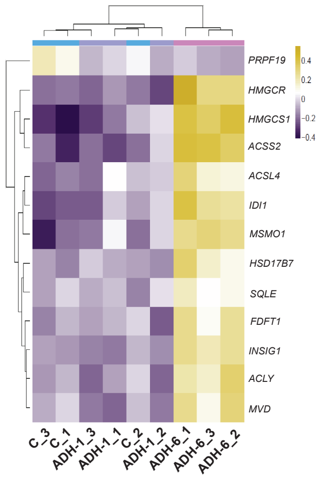

Supplementary Figure 16. Determination of the best condition for differential gene expression analysis. (**a**) Principal component analysis (PCA) illustrating total transformed variances including PC1 (36%) and PC2 (29%) for the vehicle-treated controls (C) and ADH-1 and ADH-6 treated cells. (**b**) The number of differentially expressed genes (DEGs) for comparative pairwise analysis for ADH-6/ADH-1, ADH-6/C and ADH-1/C based on statistical significance cut-offs of *P*-adj < 0.05, < 0.01 and < 0.001. Significance was assessed by false discovery rate (FDR) adjusted *P*-value (*P*-adj or q-value), which was obtained from the hypergeometric *P*-value that was corrected for multiple hypothesis testing using the Benjamin and Hochberg procedure[29]. (**c**) Gene ontology (GO) term analysis (biological processes) based on ADH-6/C (*P*-value < 0.05) displaying enrichments involved in cholesterol, lipid and isoprenoid biosynthetic processes. Statistical analysis was performed using two-tailed unpaired *t*-test. (**d**) A heatmap (scaled to log2cpm_voom) displaying expression patterns of all 15 DEGs from the GO term analysis in (**c**).

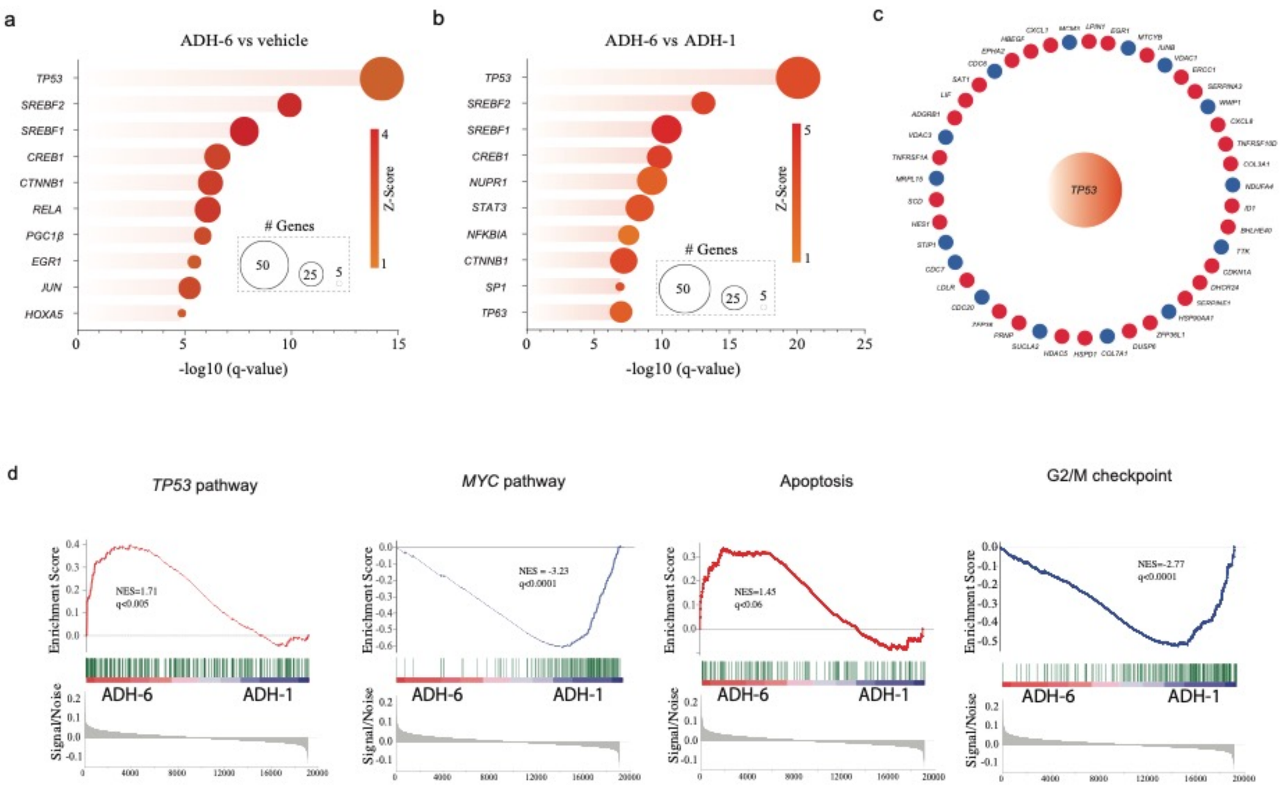

Supplementary Figure 17. Identification of transcriptional regulators of dysregulated genes in oligopyridylamide-treated MIA PaCa-2 cells. (**a**,**b**) Ingenuity pathway analysis (IPA) was performed on differentially expressed genes (DEGs) to identify transcriptional regulators (TRs) responsible for gene dysregulation in MIA PaCa-2 cells in ADH-6 vs control (**a**) and ADH-6 vs ADH-1 (**b**) samples. The top 10 TRs in each comparison (ranked based on q-values with a z-score cutoff of 1.4) are shown on the y-axis. The q-value (x-axis) for each TR represents the number of DEGs in the known pathway that overlapped with genes from our dataset. Circles sizes represent the subset of the DEGs with an expression pattern consistent with pathway activation. (**c**) Network plot for the genes that predict activation of the *TP53* pathway in the ADH-6 vs ADH-1 comparison. Red and blue nodes represent upregulated and downregulated genes, respectively. (**d**) Gene set enrichment analysis (GSEA) using gene expression data from ADH-6 and ADH-1 treated MIA PaCa-2 cells. The enrichment plots for *TP53* pathway, *MYC* pathway, apoptosis, and G2/M checkpoint are shown along with their normalized enrichment scores (NES) and q-values. Genes in the enrichment plots are marked with green vertical bars, and the gene enrichment scores are shown on the y-axis and ordered according to their signal/noise ratio. Upregulated pathways in the ADH-6 treatment group are represented by red lines (*TP53* and apoptosis) while downregulated pathways are represented by blue lines (*MYC* and G2/M).

4.3. Supplementary proteome analysis

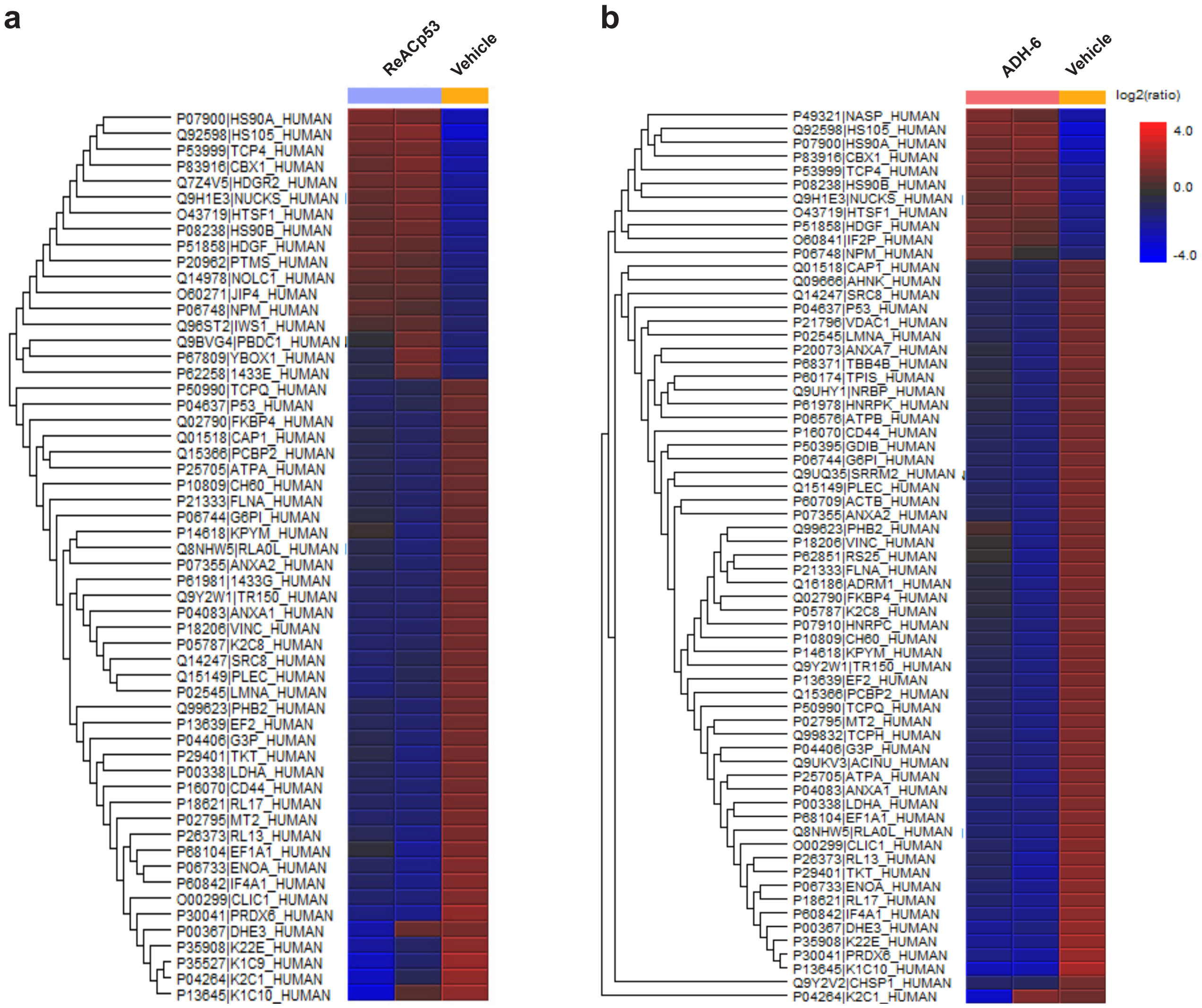

Supplementary Figure 18. Phosphoproteome analysis of ADH-6 treated MIA PaCa-2 cells. Heat map representation of the identified proteins in the vehicle (control), ADH-1, ADH-6 and ReACp53 treated groups and their corresponding protein abundances. The digests were phosphopeptide enriched using titanium dioxide (TiO_2), tandem mass tag (TMT) labeled, combined in equal amounts (1:1), and analysed by liquid chromatography tandem mass spectrometry (LC-MS/MS). Reporter ion intensities in the MS^2 spectra for the TMT labeled phosphopeptides were used to quantify protein abundance. As shown in the color scale bar, red indicates proteins that are upregulated, while dark blue colour signifies proteins that are downregulated.

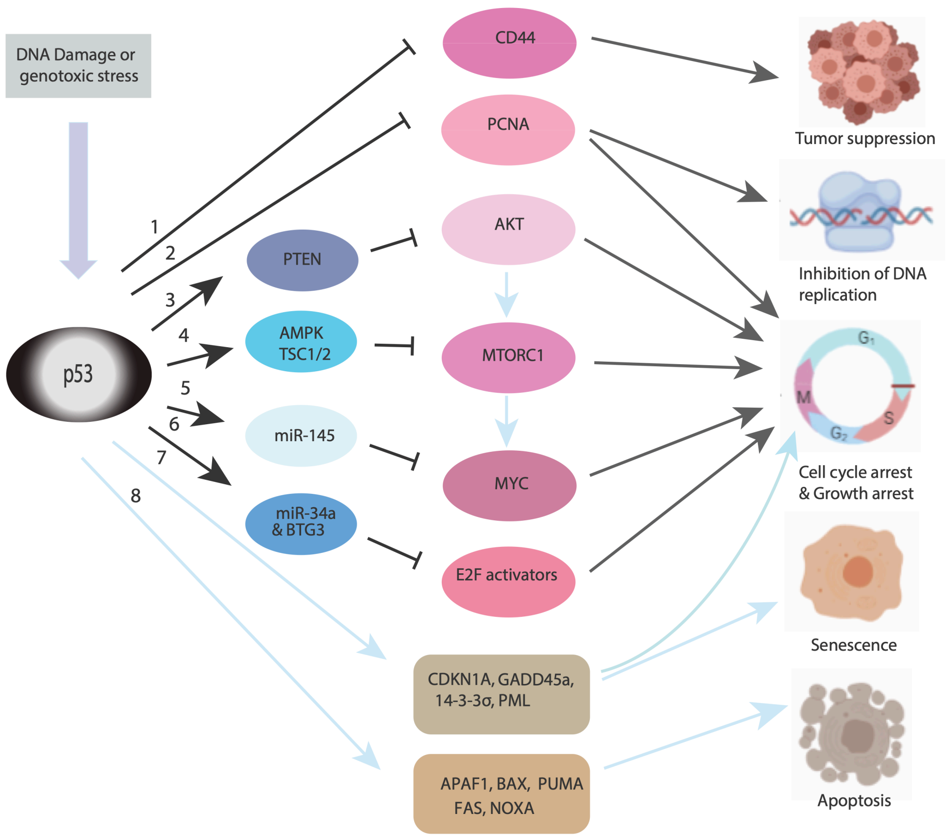

Supplementary Figure 19. A simplified model of p53-mediated regulation of DNA replication/ repair and cell cycle progression/proliferation. (**1**) p53 is reported to suppress the expression of CD44 (cluster of differentiation 44), a pro-survival protein, by binding to its promoter[30]. (**2**) Elevated expression of p53 downregulates PCNA (proliferating cell nuclear antigen) leading to the inhibition of DNA replication and cell cycle progression[31,32]. (**3**,**4**) DNA damage induced p53 expression is reported to suppress mTORC1 (mechanistic target of rapamycin complex 1) through AMPK (5′ adenosine monophosphate-activated kinase) and REDD1 (regulated in development and DNA damage 1) responses via the TSC1/2 (tuberous sclerosis proteins 1 and 2) complex and through the transactivation of PTEN (phosphatase and tensin homolog)[33–35]. (**5**) p53 induces the expression of the tumor suppressor microRNA, miR-145, which represses expression of c-Myc (avian myelocytomatosis virus oncogene cellular homolog) post-transcriptionally[36,37]. (**6**) Stress induced p53 represses the E2F (E2 promoter binding factor, an activator) pathway by inducing miR-34 and *BTG3* (B-cell translocation gene 3)[38–40]. The downregulation of proliferative pathways (**2**–**6**) results in cell cycle arrest (black arrows). (**7**,**8**) Under stress, p53 also induces the transactivation of genes that induce senescence and cause cell death[41]. Adapted from "P53 Regulation and Signalling", by BioRender.com (2020). Retrieved from https://app.biorender.com/biorender-templates.

Supplementary Table 2. Biological roles of downregulated/upregulated phosphoproteins in DNA repair/replication and cell cycle progression/proliferation

Supplementary Table 2a. Downregulated phosphoproteins

Protein	Access. Nº	Phosphory-lation site(s)	Implicated pathway(s)	Phosphorylating enzymes	Cellular function(s)	Refs	Cell line(s) used for studies
Heterogeneous nuclear ribonucleo-protein D (HNRNPD)	Q14103	Ser83	MYC, E2F & G2M checkpoint	?	Phosphorylated HNRNPD interacts and co-localizes in the cytoplasm with 14-3-3ζ. Cytoplasmic AUDF1 decreases the stability of cyclin-dependent kinase inhibitors which promotes cell proliferation. (**Cell proliferation**)	42,43	Oral squamous cell carcinoma (OSCC) cells & human thyroid carcinoma cells
Minichromosome maintenance complex component 2 (MCM2)	P49736	Ser27, Ser41 & Ser139	MYC, E2F, G2M checkpoint & MTORC1 signaling	Cdc7/Dbf4	During cell cycle, phosphorylated MCM2 is proposed to be important for regulation of ATPase activity of MCM2 and initiation of DNA replication. (**DNA repair/replication**)	44,45	HeLa cells
Lactate dehydrogenase A (LDH-A)	P00338	Y10 & Y83	MYC, oxidative phosphoryl ation, glycolysis & MTORC1	Receptor tyrosine kinase FGFR1	Phosphorylation at Y10 regulates the activity of LDH-A by increasing the formation of active, tetrameric LDH-A, while phosphorylation at Y83 mediates its binding to its substrate to promote tumor growth. (**Cell proliferation**)	46	Various human cancer cell lines, including H1299
Proliferating cell nuclear antigen (PCNA)	P12004	Y211	MYC, E2F & P53	EGF receptor	The chromatin-bound form of PCNA is phosphorylated at Y211 during the S phase of cell cycle to promote DNA replication. (**DNA repair/replication**)	47,48	Breast cancer MDA-MB-231cells & epidermoid carcinoma A431 cells
Voltage-dependent anion channel 1 (VDAC1)	P21796	S193	MYC & oxidative phosphory-lation	Nek1/PKC	Phosphorylation of VDAC1 is reported to prevent mitochondria-mediated apoptosis. (**Cell proliferation**)	49,50	Human kidney 2 (HK2) cells
Prohibitin 2 (PHB2)	Q99623	Ser91 & Ser176	MYC & oxidative phosphory-lation	Akt	Phosphorylated PHB2 in leukemia cells is proposed to regulate coordinated nuclear and mitochondrial responses, which increase cell survival. (**Cell proliferation**)	51,52	NB4 human leukemia cells
Poly(rC) binding protein 1 (PCBP1)	Q15365	Ser43	MYC	Akt2	TGF-β mediates PCBP1 phosphorylation, which promotes epithelial to mesenchymal transition (EMT) in cells that, in turn, contributes to cancer progression.	53–55	Non-small cell lung cancer (NSCLC) A549 cells & gallbladder carcinoma GBC-SD cells
		Thr60 & Thr127		p21-activated Kinase 1 (Pak1)	Phosphorylated PCBP1 causes the transactivation of eIF4E, leading to initiation of translation to promote cell growth. (**Cell proliferation**)	56,57	HeLa cells
Protein kinase, DNA-activated, catalytic subunit (PRKDC)	P78527	S2056, S2609, T2647 & T3950	E2F	Autophosphory-lation	During mitosis phosphorylated PRKDC is required for chromosome segregation and cell cycle progression. (**Cell proliferation**)	58,59	HeLa & HCT116 cells
		S3205		Polo-like kinase 1 (PLK1)	Phosphorylation of PRKDC during mitosis is required for proper cytokinesis. (**Cell proliferation**)	59,60	HeLa cells

		Thr-2609, Ser2612, Thr2638 & Thr2647		Autophosphory-lation	Phosphorylation of PRKDC leads to conformational changes within the protein that results in efficient DNA end processing and double strand break repair. (**DNA repair/replication**)	61,62	Human lymphoblastoid cells
Tripartite motif containing 28 (TRIM28)	Q13263	Ser824	MYC	ATM & DNA-PK	During DNA damage, phosphorylated TRIM28 results in de-repression of p21 and Gadd45, which causes cell cycle arrest to allow DNA repair. (**DNA repair/replication**) In cancer cells, phosphorylated TRIM28 promotes tumor growth by increasing DNA damage repair. (**Cell proliferation**)	63–66	HEK293 cells & A375 melanoma cells
		Ser473		PKCδ	During S phase, phosphorylation of TRIM28 removes its repression on Cyclin A2, which leads to cell cycle progression. (**Cell proliferation**)	67	HEK293 cells
		Tyr449, Tyr458 & Tyr517		Src family of kinases	Phosphorylation inhibits TRIM28's interaction with HP1 and heterochromatin, resulting in a loss of their transcriptional repression activity. It is speculated that TRIM28 enhances oncogenic transformation of cells. (**Cell proliferation**)	68,69	COS-1 & HeLa S3 cells
Heterogeneous nuclear ribonucleo-proteins C (HNRNPC)	P07910	Ser240, Ser225 & Ser228	MYC	CK1α	At physiological levels of H_2O_2, phosphorylated HNRNPC is predicted to mediate cell proliferation and survival. (**Cell proliferation**)	70	Human umbilical vein endothelial cells (HUVECs)
RACK1	P63244	Tyr 228 &/or Tyr246	MYC & p53	Src	SRC-mediated phosphorylation and interaction with RACK1 is predicted to enhance cell survival, proliferation and migration. (**Cell proliferation**)	71,72	NIH3T3 cells
Fas cell surface death receptor (FAS)	P49327	Y232 & Y291	Apoptosis, p53, IL6/JAK/ STAT3 & EMT	Src & Yes	Phosphorylation of the Fas death domain results in suppression of apoptosis. (**Cell proliferation**)	73,74	Breast, ovarian & colorectal cancer cell lines
Lamin A/C (LMNA)	P02545	Thr19, Ser22 & Ser392	Apoptosis	Cdk group	Phosphorylation of Lamin A/C during late mitosis results in its depolymerization of nuclear lamina, which promotes cell cycle progression. (**Cell proliferation**)	75,76	HeLa cells
ATP citrate lyase (ACLY)	p53396	Ser454	MTORC1	Akt/SREBP	Phosphorylation of ACLY is predicted to regulate cell growth and differentiation by mediating de novo lipogenesis (DNL). (**Cell proliferation**)	77,78	Adipocytes & myrAkt-ER cells
CD44	p16070	Ser325	Apoptosis, IL6/JAK/ STAT3 & EMT	Ca^{2+}/calmodulin-dependent protein kinase II (CaMKII)	Phosphorylated CD44 recruits ezrin protein, which links it to the actin cytoskeleton and is predicted to promote cell proliferation and invasiveness (**Cell proliferation**)	79–81	Flow2000 fibroblasts

Annexin A1 (ANAX1)	p04083	Ser5	Apoptosis	TRPM7/Chak1	Phosphorylated Annexin is predicted to form the complex TRPM7/Annexin A1/Mg^{2+}, which is proposed to enhance cell viability and cell cycle progression. (**Cell proliferation**)	82,83	Human vascular & smooth muscle cells
RAB1A, member RAS oncogene family (RAB1A)	p62820	Ser194	MTORC1	Cdk1	During mitosis, to ensure equal distribution of organelles between daughter cells, phosphorylation of RAB1A inhibits intracellular transport. (**Cell proliferation**)	84,85	HeLa cells

Supplementary Table 2b. Upregulated phosphoproteins

Protein	Access. Nº	Phosphory-lation site(s)	Implicated pathway(s)	Phosphorylating enzymes	Cellular function(s)	Refs	Cell line(s) used for studies
Splicing factor 3b subunit 2 (SF3B2)	Q13435	Ser289	DNA damage repair	ATM	During cell cycle, phosphorylated SF3B2 recruits CtIP at double stranded DNA breaks. CtIP also promotes p21 and Gadd45 causing cell cycle arrest. (**DNA repair/replication**)	86–88	U2OS cells
Vacuolar protein sorting 4 homolog B (VPS4B)	O75351	Ser102 & Ser108	Multi-vesicular body (MVB) sorting	CK2α pathway	Phosphorylated VPS4B, along with ESCRTIII subunits, regulates epidermal growth factor degradation through MVB sorting pathway. This is predicted to inhibit cell proliferation. (**Cell proliferation**)	89,90	HEK 293T & HeLa cells

SECTION 5. Supplementary *in vivo* tumor reduction data

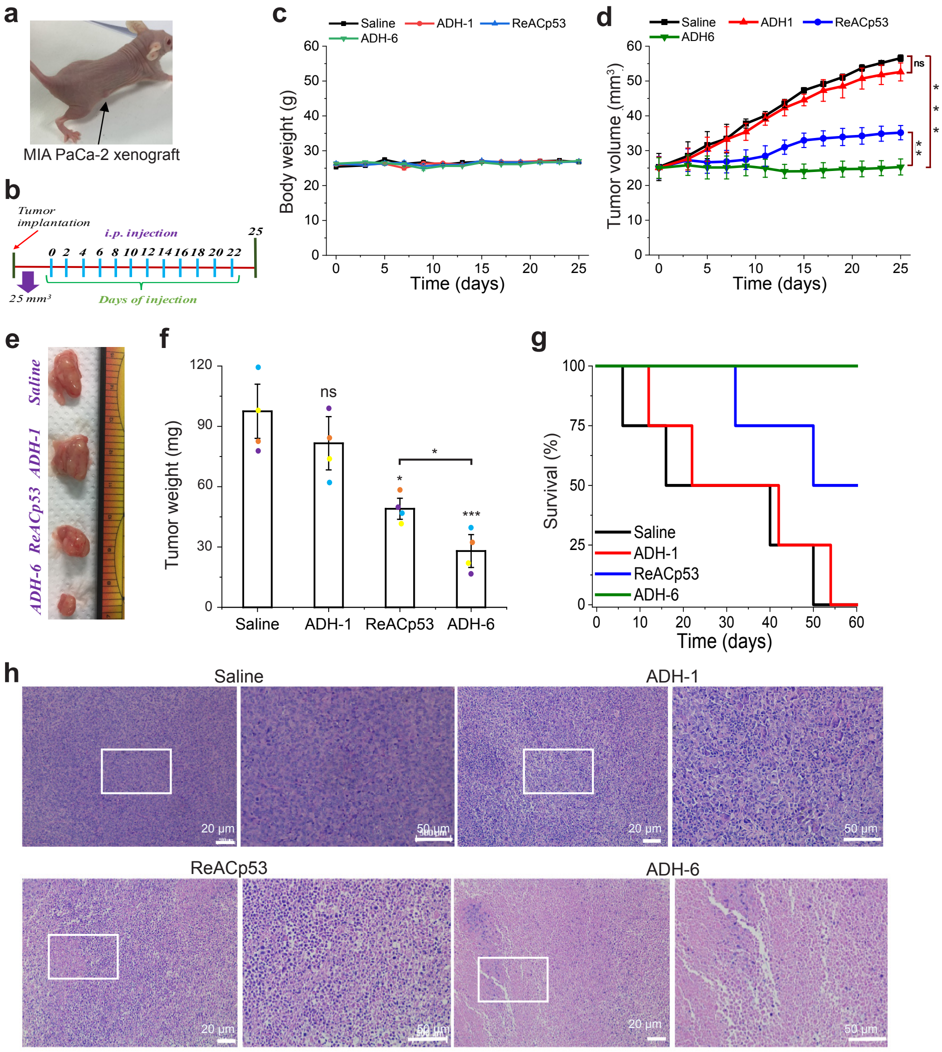

Supplementary Figure 20. Effects of ADH-6 on MIA PaCa-2 xenografts *in vivo*. (**a,b**) Design of the tumor reduction studies. A representative mouse bearing MIA PaCa-2 (mutant R248W p53) xenograft (**a**) and the treatment schedule (**b**). Once the tumor volume reached ~25 mm^3, the mice were randomized into the different treatment groups ($n = 8$ per group), which were injected intraperitoneally with saline or 156.4 μM ADH-1, ReACp53 or ADH-6. Injections were done every 2 days for a total of 12 doses, with the first day of treatment defined as day 0. (**c**) Body weight changes of the tumor-bearing mice in the different treatment groups ($n = 8$ per group) monitored for the duration of the experiment. Data presented are mean ± SD. (**d**) Tumor volume growth curves for the MIA PaCa-2 xenografts in the different treatment groups over 25 days of treatment (mean ± SD; n = 8). Statistical analysis was performed using one-way ANOVA followed by Tukey's *post hoc* test. P = 0.0005 for ADH-6 vs saline, and P = 0.0012 for ADH-6 vs ReACp53. (**e,f**) Tumor mass analysis for the different treatment groups. After 25 days of treatment, 4 mice per treatment group were sacrificed and the tumor tissues were isolated and imaged (**e**) and subsequently weighed to determine the tumor mass (**f**). Data presented are mean ± SD, and statistical analysis was performed using one-way ANOVA followed by Tukey's *post hoc* test. P = 0.0004 for ADH-6 vs saline, P = 0.0223 for ReACp53 vs saline, P = 0.033 and ADH-6 vs ReACp53. (**g**) Survival curves for the saline, ADH-1, ReACp53 and ADH-6 treatment groups over 60 days (n = 4 per group). (**h**) Hematoxylin and eosin (H&E)-stained xenograft sections from the different treatment groups following 25 days of treatment. Images shown are representative of xenograft sections from four mice per treatment group. Images on the right are magnified views of the boxed regions in the images on the left. Scale bar = 20 μm (50 μm for the magnified views). *$P < 0.05$, **$P < 0.01$, ***$P < 0.001$ or non-significant (ns, $P > 0.05$) for comparisons with controls and amongst the different treatment groups.

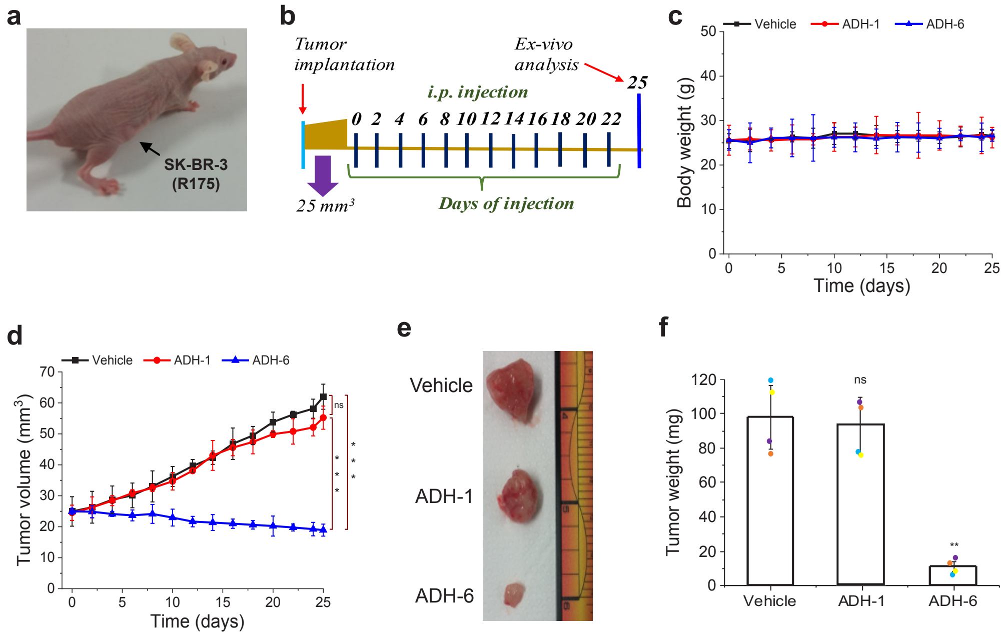

Supplementary Figure 21. Effects of ADH-6 on SK-BR-3 xenografts *in vivo*. (**a,b**) A representative mouse bearing SK-BR-3 (mutant R175H p53) xenograft (**a**) and the treatment schedule (**b**). Once the tumor volume reached ~25 mm^3, the mice were randomized into the different treatment groups ($n = 8$ per group), which were injected intraperitoneally with vehicle (0.02% DMSO) or 716.4 μM ADH-1 or ADH-6. Injections were done every 2 days for a total of 12 doses, with the first day of treatment defined as day 0. (**c**) Body weight changes of the tumor-bearing mice in the different treatment groups ($n = 8$ per group) monitored for the duration of the experiment. Data presented are mean ± SD. (**d**) Tumor volume growth curves for the SK-BR-3 xenografts in the different treatment groups over 25 days of treatment (mean ± SD; $n = 8$). Statistical analysis was performed using one-way ANOVA followed by Tukey's *post hoc* test. $P < 0.0001$ for ADH-6 vs vehicle, and $P = 0.0007$ for ADH-6 vs ADH-1. (**e,f**) Tumor mass analysis for the different treatment groups. After 25 days of treatment, 4 mice per treatment group were sacrificed and the tumor tissues were isolated and imaged (**e**) and subsequently weighed to determine the tumor mass (**f**). Data presented are mean ± SD, and statistical analysis was performed using one-way ANOVA followed by Tukey's *post hoc* test. $P = 0.0026$ for ADH-6 vs vehicle. $^{**}P < 0.01$, $^{***}P < 0.001$ or non-significant (ns, $P > 0.05$) for comparisons with controls and amongst the different treatment groups.

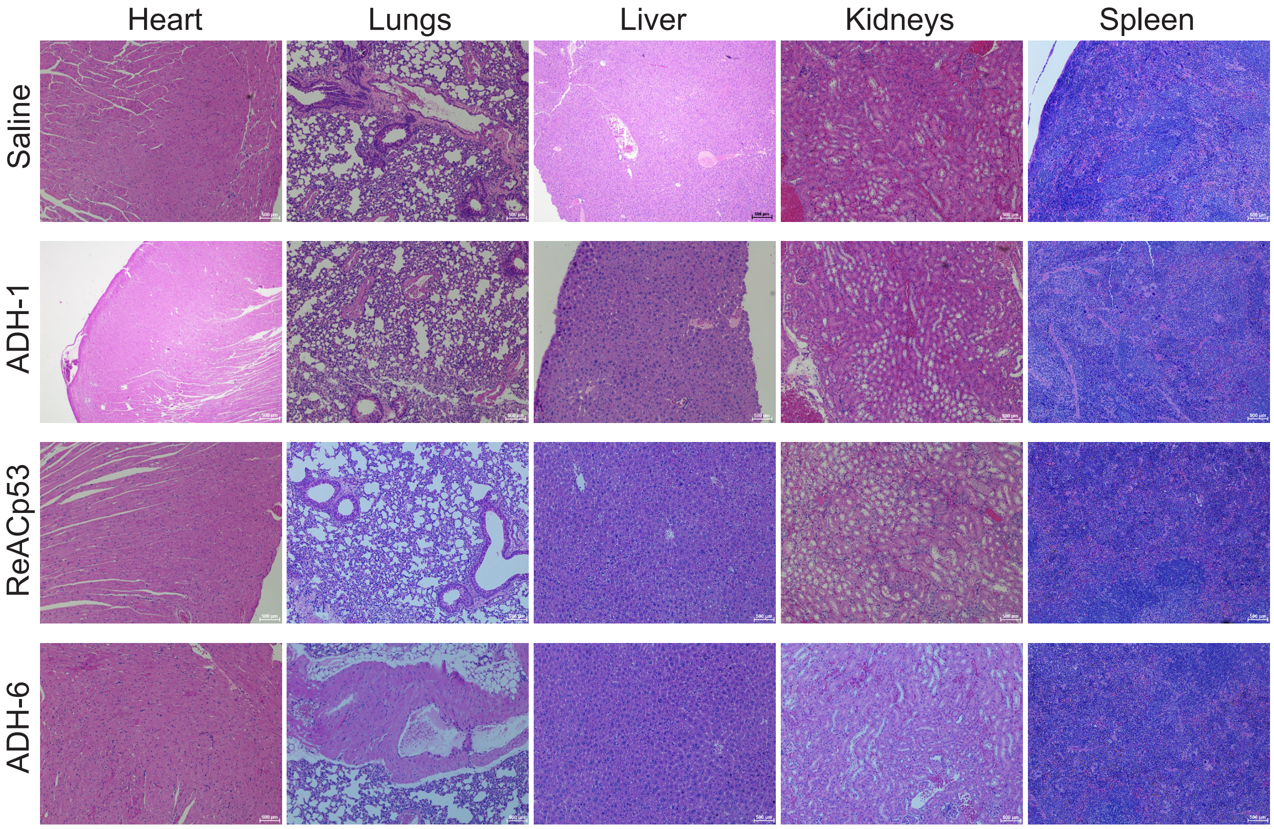

Supplementary Figure 22. Histological analysis of vital organs following treatment with lower doses of ADH-6. Hematoxylin and eosin (H&E) staining of heart, lung, liver, kidney and spleen sections from MIA PaCa-2 tumor-bearing mice after 25 days of treatment with saline, ADH-1, ReACp53 or ADH-6 (dosage of 155.6 µM in saline, administered every 2 days, for a total of 12 doses). Images shown are representative of tissue sections from four mice per treatment group. Scale bar = 50 µm.

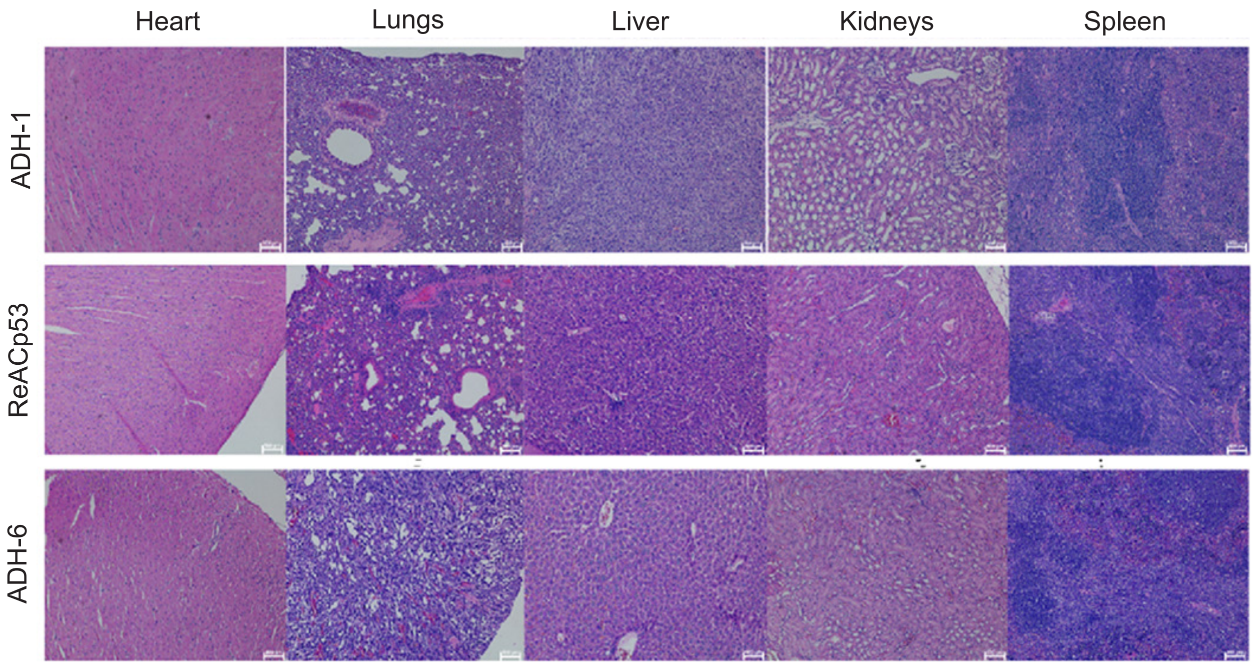

Supplementary Figure 23. Histological analysis of vital organs following treatment with higher doses of ADH-6. Hematoxylin and eosin (H&E) staining of heart, lung, liver, kidney and spleen sections from MIA PaCa-2 tumor-bearing mice after 25 days of treatment with ADH-1, ReACp53 or ADH-6 (dosage of 716.4 µM in 0.02% DMSO, administered every 2 days, for a total of 12 doses). Images shown are representative of tissue sections from four mice per treatment group. Scale bar = 50 µm.

SECTION 6. Synthesis and characterization of ADH-6

The synthesis and characterization of the monomer pyridyls have been reported previously[91].

General method for the reduction of arylamides

To a solution of nitro arylamide (0.1 mmol) in EtOAc (10 mL), Pd/C (10% by wt) was added and the reaction started with constant stirring in an H_2 (g) atmosphere at room temperature. The progress of the reaction was monitored using thin layer chromatography (TLC). The disappearance of the starting material confirmed the completion of the reaction. The reaction mixture was filtered and the filtrate was dried on a rotovap to afford the desired product as a yellow solid, which was used in the next step without further characterization.

General method for the deprotection of oligopyridylamides

To a solution of the oligopyridylamide (50 μmol) in dichloromethane (5 mL), triethylsilane (250 μL) was added, followed by addition of trifluoroacetic acid (TFA, 500 μL), and the reaction solution was stirred constantly for 4 h. The reaction solution was dried on a rotovap and washed with cold diethyl ether (3×5 mL) which resulted in a yellow powder.

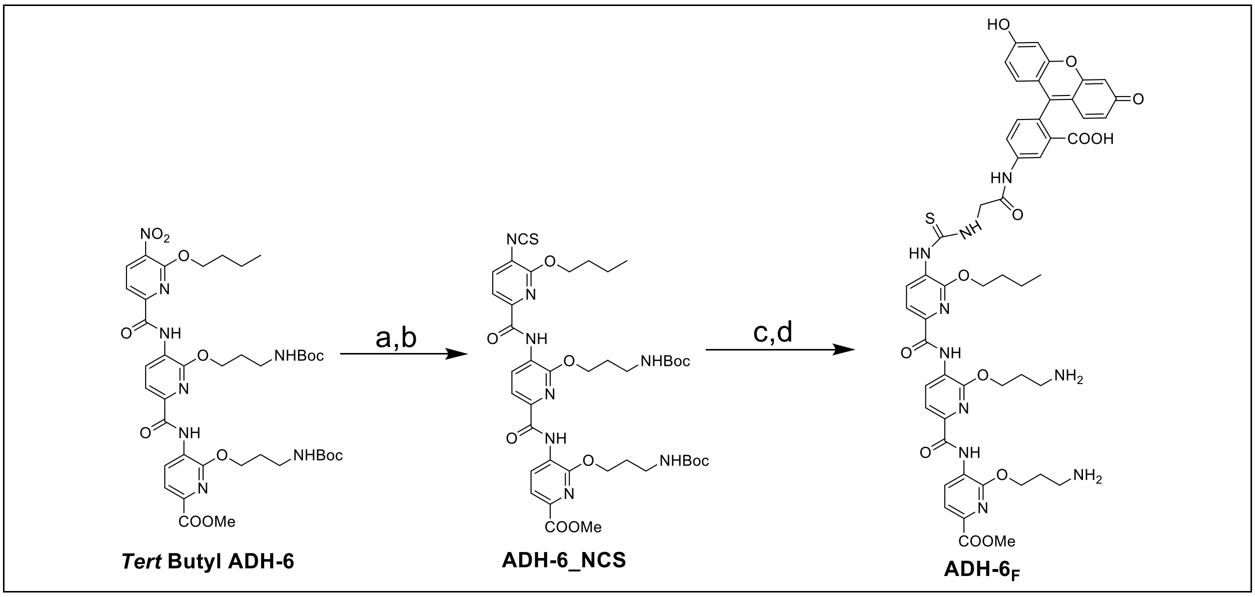

Scheme S1. Synthetic route for the synthesis of ADH-6$_F$ (ADH-6$_{FITC}$).

ADH-6

The synthesis and characterization of ADH-6 (previously identified as ADH-40) was reported elsewhere[92].

ADH-6-NH_2

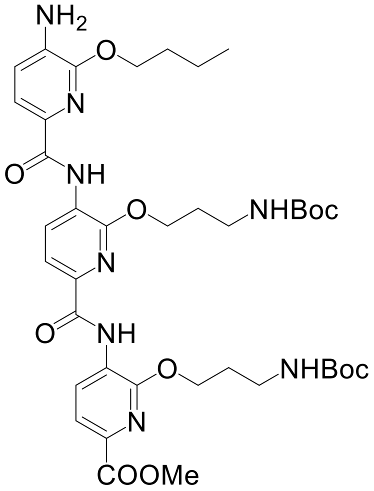

To a solution of ADH-6 (0.1 mmol) in EtOAc (10 mL), Pd/C (12% by wt) was added and the reaction started with constant stirring in an H_2 (g) atmosphere at room temperature. The progress of the reaction was monitored using TLC. The disappearance of the starting material confirmed the completion of the reaction (~4 h). The reaction mixture was filtered, and the filtrate was dried on a rotovap to afford the desired product as a yellow solid (yield = 86%), which was used in the next step without further characterization.

ADH-6-NCS

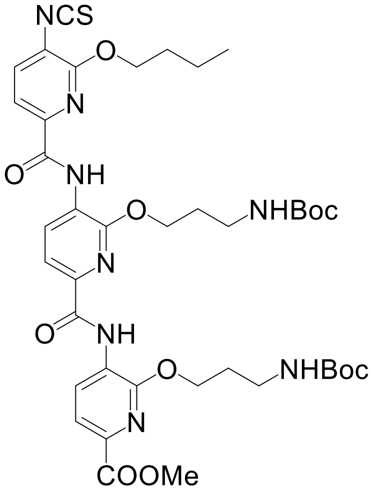

To a solution of **ADH-6-NH_2** (40 mg, 0.049 mmol) was dissolved in dicholoromethane (anhydrous, 10 mL), followed by the addition of 1,1'-Thiocarbonyldi-2(1H)-pyridone (22.7 mg, 0.098 mmol, 2 eq.) and the reaction solution was stirred for 6 h at room temperature under inert atmosphere. The progress of the reaction was monitored by TLC. Flash chromatography (0 to 60% Ethyl acetate in hexane) yielded the desired product as a yellow solid (36.6 mg, 87%). ^{1}H NMR (600 MHz, Chloroform-*d*) δ ^{1}H NMR (600 MHz, Chloroform-*d*) δ 10.32 – 10.28 (s, 1H), 10.25 – 10.22 (s, 1H), 9.04 – 9.00 (d, J = 8.0 Hz, 1H), 8.94 – 8.90 (d, J = 8.0 Hz, 1H), 8.47 – 8.42 (d, J = 7.8 Hz, 1H), 8.04 – 7.99 (d, J = 8.0 Hz, 2H), 7.88 – 7.83 (d, J = 8.0 Hz, 1H), 4.72 – 4.67 (t, J = 6.3 Hz, 2H), 4.66 – 4.62 (t, J = 6.2 Hz, 4H), 4.13 – 3.85 (s, 3H), 3.44 – 3.38 (q, J = 6.2 Hz, 2H), 3.32 – 3.28 (m, 2H), 2.20 – 2.12 (p, J = 6.6 Hz, 2H), 2.12 – 2.03 (h, J = 5.2, 3.8 Hz, 2H), 1.97 – 1.90 (m, 2H), 1.71 – 1.59 (m, 5H), 1.49 – 1.46 (s, 9H), 1.41 – 1.37 (s, 9H). MS-ESI (*m/z*): calculated for $C_{40}H_{52}N_8O_{11}S$ (M): 852.9610, found 852.9689.

***Tert* Butyl ADH-6$_F$**

To a solution of **ADH-6-NCS** (25 mg, 0.029 mmol) in pyridine (5 ml, anhydrous), N, N-diisopropylethylamine (0.005 ml, 0.05 mmol) was added and the solution was stirred for 10 min. To this solution, 5-(aminoacetamido) fluorescein (23.9 mg, 0.056 mmol) was added and the reaction was started in dark with continuous stirring under an inert atmosphere. The reaction solution was stirred overnight in the dark. The product was purified using column chromatography (0–20% methanol in dichloromethane with 1% triethylamine, v/v) as an orange solid (20 mg, 54%). The compound (*tert*-butyl ADH-6$_F$) was characterized via MALDI-TOF and used in the next step without any further characterization. The ^{1}H NMR peaks were very broad potentially because of the stacking of the molecule. We used the compound in the next step without further characterization.

ADH-6$_F$ (ADH-6$_{FITC}$)

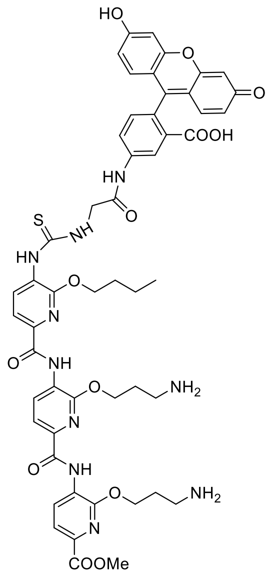

To a solution of **tert-butyl ADH-6$_F$** (17 mg, 0.014 mmol) in dichloromethane (4 mL), triethylsilane (0.1 mL, excess) was added, followed by the addition of TFA (0.4 mL, excess) and the reaction solution was stirred in the dark at room temperature for 4 h. The solution was then dried, and the orange solid was washed with cold diethyl ether (3×5 mL), which afforded the desired product (ADH-6$_F$) as an orange solid. The product was further reconstituted in DMSO/water (75:25, v/v) and purified on a semi-preparative reverse-phase HPLC. Solvents A (95% water, 5% ACN and 0.1% TFA) and B (95% ACN, 5% water and 0.1% TFA) were used for the purification. The desired product (ADH-6$_F$) was obtained as an orange solid (11.8 mg, 80%). ^{1}H NMR (600 MHz, DMSO-d_6) δ 10.78 – 10.70 (s, 1H), 10.29 – 10.27 (s, 1H), 10.26 – 10.23 (m, 1H), 10.17 – 10.11 (s, 2H), 9.71 – 9.60 (s, 1H), 9.03 – 8.99 (d, *J* = 8.0 Hz, 1H), 8.95 – 8.89 (t, *J* = 6.6 Hz, 1H), 8.86 – 8.78 (d, *J* = 7.9 Hz, 1H), 8.41 – 8.33 (s, 1H), 8.00 – 7.95 (dd, *J* = 8.1, 2.9 Hz, 1H), 7.30 – 7.22 (d, *J* = 8.2 Hz, 1H), 6.70 – 6.67 (d, *J* = 2.2 Hz, 2H), 6.63 – 6.58 (d, *J* = 8.5 Hz, 2H), 6.57 – 6.52 (d, *J* = 7.8 Hz, 4H), 4.70 – 4.64 (t, *J* = 5.8 Hz, 2H), 4.63 – 4.58 (t, *J* = 6.3 Hz, 2H), 4.58 – 4.53 (q, *J* = 7.3, 6.8 Hz, 2H), 4.52 – 4.48 (d, *J* = 4.9 Hz, 2H), 3.98 – 3.78 (s, 3H), 3.08 – 3.01 (m, 6H), 2.24 – 2.18 (p, *J* = 7.5, 7.0 Hz, 2H), 2.18 – 2.11 (p, *J* = 6.9 Hz, 2H), 1.97 – 1.85 (h, *J* = 6.9 Hz, 2H), 1.64 – 1.51 (dh, *J* = 14.4, 7.4 Hz, 3H). MS-ESI (*m/z*): calculated for $C_{55}H_{50}N_{10}O_{13}S$ (M+H): 1058.1130, found 1058.1148.

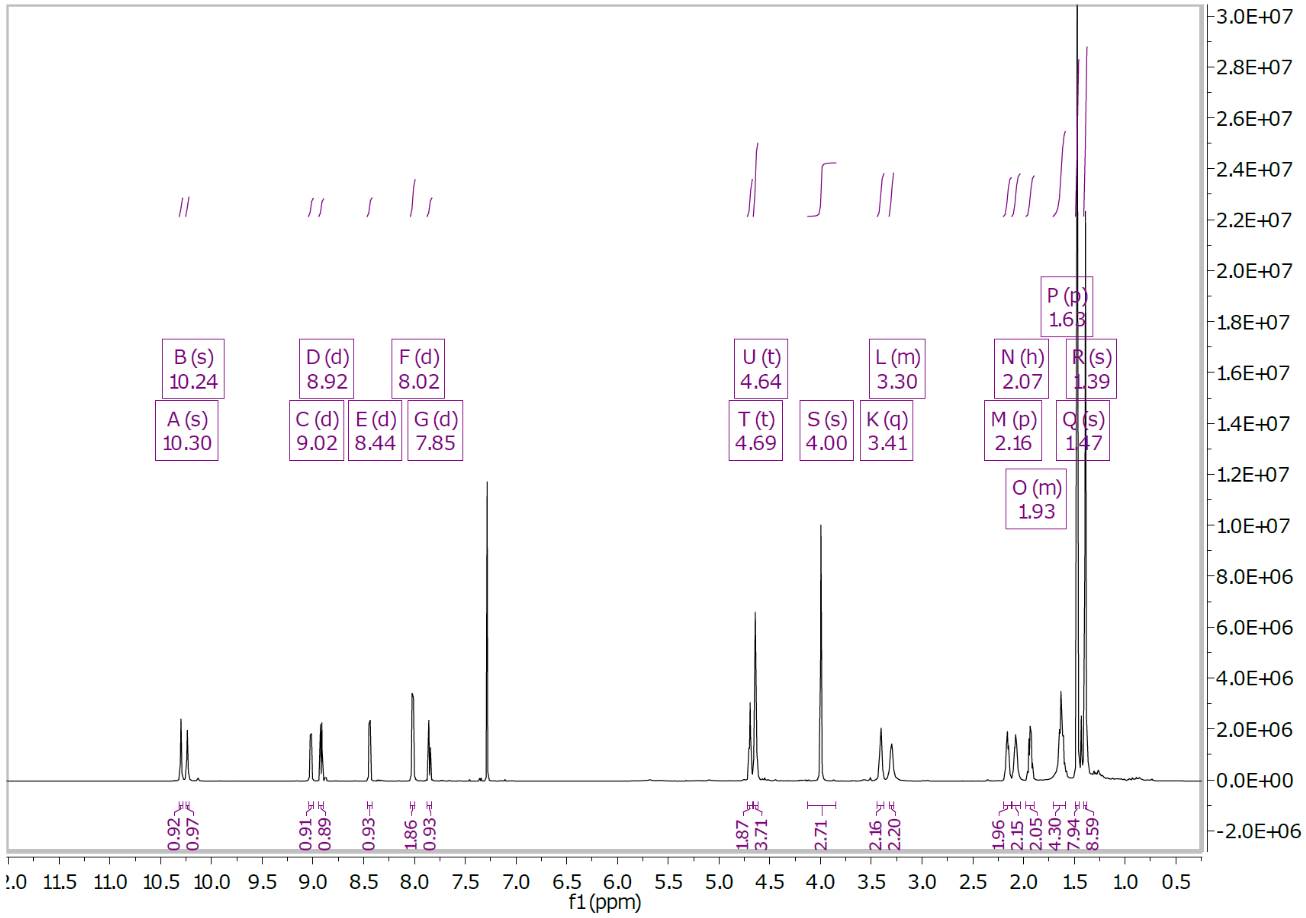

Supplementary Figure 24. ^{1}H NMR of ADH-6-NCS.

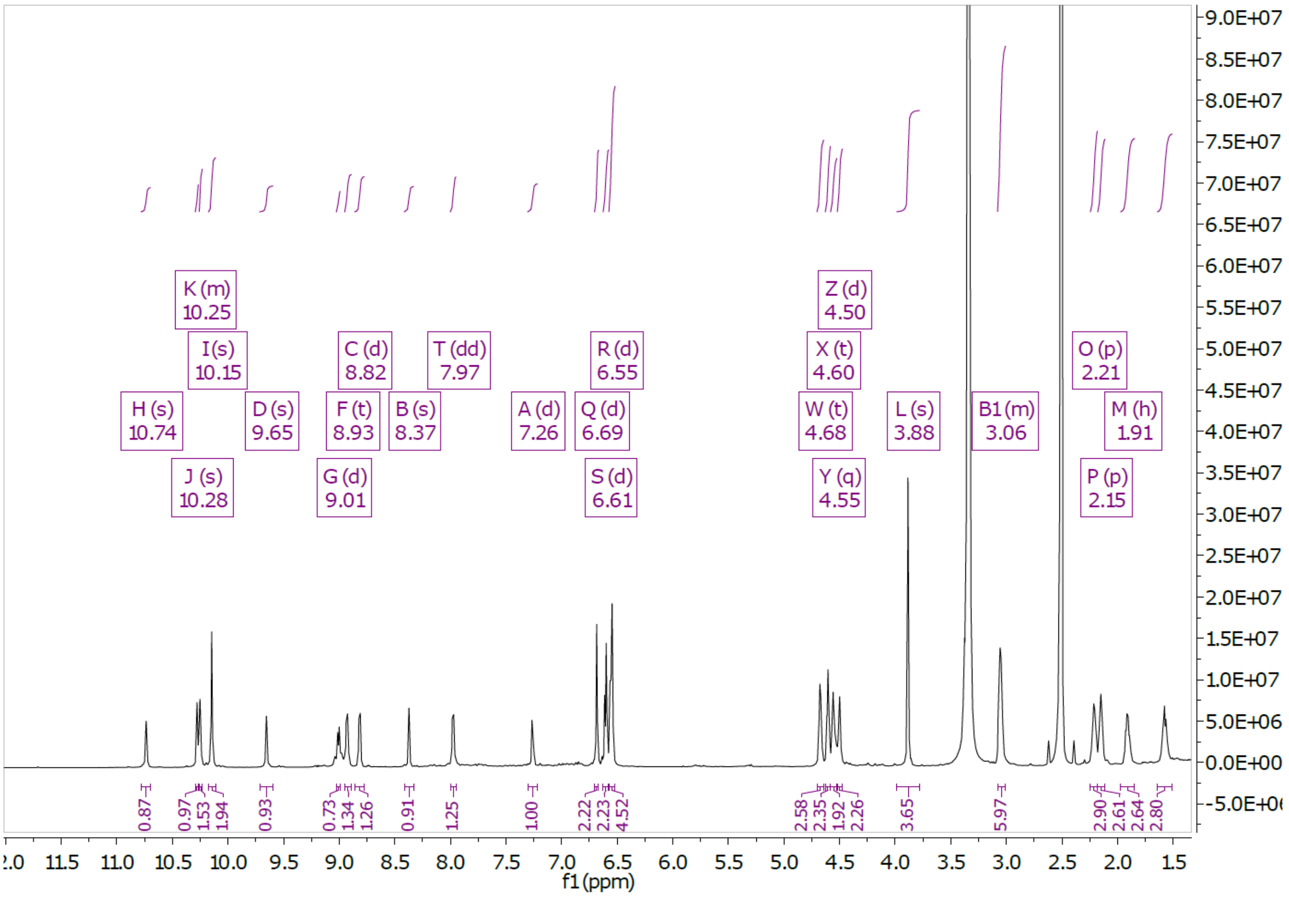

Supplementary Figure 25. **^{1}H NMR of ADH-6$_F$ (ADH-6$_{FITC}$).**

SUPPLEMENTARY REFERENCES

1. Rasquinha, J. A., Bej, A., Dutta, S. & Mukherjee, S. Intrinsic Differences in Backbone Dynamics between Wild Type and DNA-Contact Mutants of the p53 DNA Binding Domain Revealed by Nuclear Magnetic Resonance Spectroscopy. *Biochemistry* **56**, 4962–4971 (2017).
2. Cañadillas, J. M. P. *et al.* Solution structure of p53 core domain: Structural basis for its instability. *PNAS* **103**, 2109–2114 (2006).
3. Dunn, K. W., Kamocka, M. M. & and McDonald, J. H. A practical guide to evaluating colocalization in biological microscopy. *American Journal of Physiology - Cell Physiology* **300**, 723–742 (2011).
4. Abbas, T. & Dutta, A. p21 in cancer: intricate networks and multiple activities. *Nat. Rev. Cancer* **9**, 400–414 (2009).
5. Karimian, A., Ahmadi, Y. & Yousefi, B. Multiple functions of p21 in cell cycle, apoptosis and transcriptional regulation after DNA damage. *DNA Repair (Amst.)* **42**, 63–71 (2016).
6. Polyak, K., Xia, Y., Zweier, J. L., Kinzler, K. W. & Vogelstein, B. A model for p53-induced apoptosis. *Nature* **389**, 300–305 (1997).
7. Lee, J.-H. *et al.* The p53-inducible gene 3 (PIG3) contributes to early cellular response to DNA damage. *Oncogene* **29**, 1431–1450 (2010).
8. Aubrey, B. J., Kelly, G. L., Janic, A., Herold, M. J. & Strasser, A. How does p53 induce apoptosis and how does this relate to p53-mediated tumour suppression? *Cell Death & Differentiation* **25**, 104–113 (2018).
9. Albert, M.-C., Brinkmann, K. & Kashkar, H. Noxa and cancer therapy. *Mol Cell Oncol* **1**, (2014).
10. Montero, J. *et al.* Destabilization of NOXA mRNA as a common resistance mechanism to targeted therapies. *Nature Communications* **10**, 5157 (2019).
11. Oda, E. *et al.* Noxa, a BH3-only member of the Bcl-2 family and candidate mediator of p53-induced apoptosis. *Science* **288**, 1053–1058 (2000).
12. Kaeser, M. D. & Iggo, R. D. Chromatin immunoprecipitation analysis fails to support the latency model for regulation of p53 DNA binding activity in vivo. *Proc Natl Acad Sci U S A* **99**, 95–100 (2002).
13. Swift, J. & Coruzzi, G. A Matter of Time - How Transient Transcription Factor Interactions Create Dynamic Gene Regulatory Networks. *Biochim Biophys Acta* **1860**, 75–83 (2017).
14. el-Deiry, W. S. *et al.* WAF1, a potential mediator of p53 tumor suppression. *Cell* **75**, 817–825 (1993).
15. Miyashita, T. & Reed, J. C. Tumor suppressor p53 is a direct transcriptional activator of the human bax gene. *Cell* **80**, 293–299 (1995).
16. Nakano, K. & Vousden, K. H. PUMA, a novel proapoptotic gene, is induced by p53. *Mol Cell* **7**, 683–694 (2001).
17. Wu, X., Bayle, J. H., Olson, D. & Levine, A. J. The p53-mdm-2 autoregulatory feedback loop. *Genes Dev* **7**, 1126–1132 (1993).
18. Kastan, M. B. *et al.* A mammalian cell cycle checkpoint pathway utilizing p53 and GADD45 is defective in ataxia-telangiectasia. *Cell* **71**, 587–597 (1992).
19. Kouwenhoven, E. N. *et al.* Genome-wide profiling of p63 DNA-binding sites identifies an element that regulates gene expression during limb development in the 7q21 SHFM1 locus. *PLoS Genet* **6**, e1001065 (2010).
20. Koeppel, M. *et al.* Crosstalk between c-Jun and TAp73α/β contributes to the apoptosis–survival balance. *Nucleic Acids Res* **39**, 6069–6085 (2011).
21. Shahbazi, J., Lock, R. & Liu, T. Tumor Protein 53-Induced Nuclear Protein 1 Enhances p53 Function and Represses Tumorigenesis. *Front Genet* **4**, 80 (2013).
22. Elkeles, A. *et al.* The c-fos Proto-Oncogene Is a Target for Transactivation by the p53 Tumor Suppressor. *Mol Cell Biol* **19**, 2594–2600 (1999).
23. Yu, J., Baron, V., Mercola, D., Mustelin, T. & Adamson, E. D. A network of p73, p53 and Egr1 is required for efficient apoptosis in tumor cells. *Cell Death Differ.* **14**, 436–446 (2007).
24. Chen, C.-L., Ip, S.-M., Cheng, D., Wong, L.-C. & Ngan, H. Y. S. p73 Gene Expression in Ovarian Cancer Tissues and Cell Lines. *Clin Cancer Res* **6**, 3910–3915 (2000).

25. Soragni, A. *et al.* A Designed Inhibitor of p53 Aggregation Rescues p53 Tumor Suppression in Ovarian Carcinomas. *Cancer Cell* **29**, 90–103 (2016).
26. Towers, C. G. *et al.* The Six1 oncoprotein downregulates p53 via concomitant regulation of RPL26 and microRNA-27a-3p. *Nat Commun* **6**, (2015).
27. Mullen, P. J., Yu, R., Longo, J., Archer, M. C. & Penn, L. Z. The interplay between cell signalling and the mevalonate pathway in cancer. *Nature Reviews Cancer* **16**, 718–731 (2016).
28. Freed-Pastor, W. A. *et al.* Mutant p53 disrupts mammary tissue architecture via the mevalonate pathway. *Cell* **148**, 244–258 (2012).
29. Benjamini, Y. & Hochberg, Y. Controlling the False Discovery Rate: A Practical and Powerful Approach to Multiple Testing. *Journal of the Royal Statistical Society. Series B (Methodological)* **57**, 289–300 (1995).
30. Godar, S. *et al.* Growth-inhibitory and tumor- suppressive functions of p53 depend on its repression of CD44 expression. *Cell* **134**, 62–73 (2008).
31. Mercer, W. E., Shields, M. T., Lin, D., Appella, E. & Ullrich, S. J. Growth suppression induced by wild-type p53 protein is accompanied by selective down-regulation of proliferating-cell nuclear antigen expression. *Proc. Natl. Acad. Sci. U.S.A.* **88**, 1958–1962 (1991).
32. Shivakumar, C. V., Brown, D. R., Deb, S. & Deb, S. P. Wild-type human p53 transactivates the human proliferating cell nuclear antigen promoter. *Mol Cell Biol* **15**, 6785–6793 (1995).
33. Stambolic, V. *et al.* Regulation of PTEN transcription by p53. *Mol. Cell* **8**, 317–325 (2001).
34. Feng, Z., Zhang, H., Levine, A. J. & Jin, S. The coordinate regulation of the p53 and mTOR pathways in cells. *Proc. Natl. Acad. Sci. U.S.A.* **102**, 8204–8209 (2005).
35. Hasty, P., Sharp, Z. D., Curiel, T. J. & Campisi, J. mTORC1 and p53: clash of the gods? *Cell Cycle* **12**, 20–25 (2013).
36. Zheng, H. *et al.* Pten and p53 Converge on c-Myc to Control Differentiation, Self-renewal, and Transformation of Normal and Neoplastic Stem Cells in Glioblastoma. *Cold Spring Harb Symp Quant Biol* **73**, 427–437 (2008).
37. Sachdeva, M. *et al.* p53 represses c-Myc through induction of the tumor suppressor miR-145. *Proc. Natl. Acad. Sci. U.S.A.* **106**, 3207–3212 (2009).
38. Timmers, C. *et al.* E2f1, E2f2, and E2f3 control E2F target expression and cellular proliferation via a p53-dependent negative feedback loop. *Mol. Cell. Biol.* **27**, 65–78 (2007).
39. Welch, C., Chen, Y. & Stallings, R. L. MicroRNA-34a functions as a potential tumor suppressor by inducing apoptosis in neuroblastoma cells. *Oncogene* **26**, 5017–5022 (2007).
40. Polager, S. & Ginsberg, D. p53 and E2f: partners in life and death. *Nat. Rev. Cancer* **9**, 738–748 (2009).
41. Gatz, S. A. & Wiesmüller, L. p53 in recombination and repair. *Cell Death Differ.* **13**, 1003–1016 (2006).
42. Trojanowicz, B. *et al.* The role of AUF1 in thyroid carcinoma progression. *Endocr. Relat. Cancer* **16**, 857–871 (2009).
43. Kumar, M. *et al.* Nuclear heterogeneous nuclear ribonucleoprotein D is associated with poor prognosis and interactome analysis reveals its novel binding partners in oral cancer. *J Transl Med* **13**, 285 (2015).
44. Tsuji, T., Ficarro, S. B. & Jiang, W. Essential Role of Phosphorylation of MCM2 by Cdc7/Dbf4 in the Initiation of DNA Replication in Mammalian Cells. *Mol Biol Cell* **17**, 4459–4472 (2006).
45. Fei, L. & Xu, H. Role of MCM2–7 protein phosphorylation in human cancer cells. *Cell & Bioscience* **8**, 43 (2018).
46. Fan, J. *et al.* Tyrosine Phosphorylation of Lactate Dehydrogenase A Is Important for NADH/NAD+ Redox Homeostasis in Cancer Cells ▿ . *Mol Cell Biol* **31**, 4938–4950 (2011).
47. Wang, S.-C. *et al.* Tyrosine phosphorylation controls PCNA function through protein stability. *Nature Cell Biology* **8**, 1359–1368 (2006).
48. Zhao, H. *et al.* Interaction of proliferation cell nuclear antigen (PCNA) with c-Abl in cell proliferation and response to DNA damages in breast cancer. *PLoS ONE* **7**, e29416 (2012).
49. Chen, Y., Craigen, W. J. & Riley, D. J. Nek1 regulates cell death and mitochondrial membrane permeability through phosphorylation of VDAC1. *Cell Cycle* **8**, 257–267 (2009).

50. Kerner, J., Lee, K., Tandler, B. & Hoppel, C. L. VDAC proteomics: post-translation modifications. *Biochim. Biophys. Acta* **1818**, 1520–1525 (2012).
51. Bavelloni, A. *et al.* Prohibitin 2 represents a novel nuclear AKT substrate during all-trans retinoic acid-induced differentiation of acute promyelocytic leukemia cells. *FASEB J.* **28**, 2009–2019 (2014).
52. Peng, Y.-T., Chen, P., Ouyang, R.-Y. & Song, L. Multifaceted role of prohibitin in cell survival and apoptosis. *Apoptosis* **20**, 1135–1149 (2015).
53. Song, Q., Sheng, W., Zhang, X., Jiao, S. & Li, F. ILEI drives epithelial to mesenchymal transition and metastatic progression in the lung cancer cell line A549. *Tumour Biol.* **35**, 1377–1382 (2014).
54. Xue, X. *et al.* SchA-p85-FAK complex dictates isoform-specific activation of Akt2 and subsequent PCBP1-mediated post-transcriptional regulation of TGFβ-mediated epithelial to mesenchymal transition in human lung cancer cell line A549. *Tumour Biol.* **35**, 7853–7859 (2014).
55. Zhang, H.-Y. & Dou, K.-F. PCBP1 is an important mediator of TGF-β-induced epithelial to mesenchymal transition in gall bladder cancer cell line GBC-SD. *Mol Biol Rep* **41**, 5519–5524 (2014).
56. Meng, Q. *et al.* Signaling-dependent and coordinated regulation of transcription, splicing, and translation resides in a single coregulator, PCBP1. *PNAS* **104**, 5866–5871 (2007).
57. Huo, L.-R. & Zhong, N. Identification of transcripts and translatants targeted by overexpressed PCBP1. *Biochim. Biophys. Acta* **1784**, 1524–1533 (2008).
58. Lee, K.-J. *et al.* Involvement of DNA-dependent protein kinase in normal cell cycle progression through mitosis. *J. Biol. Chem.* **286**, 12796–12802 (2011).
59. Jette, N. & Lees-Miller, S. P. The DNA-dependent protein kinase: a multifunctional protein kinase with roles in DNA double strand break repair and mitosis. *Prog Biophys Mol Biol* **117**, 194–205 (2015).
60. Douglas, P. *et al.* Polo-like kinase 1 (PLK1) and protein phosphatase 6 (PP6) regulate DNA-dependent protein kinase catalytic subunit (DNA-PKcs) phosphorylation in mitosis. *Biosci. Rep.* **34**, (2014).
61. Douglas, P. *et al.* Identification of in vitro and in vivo phosphorylation sites in the catalytic subunit of the DNA-dependent protein kinase. *Biochem. J.* **368**, 243–251 (2002).
62. Ding, Q. *et al.* Autophosphorylation of the Catalytic Subunit of the DNA-Dependent Protein Kinase Is Required for Efficient End Processing during DNA Double-Strand Break Repair. *Mol Cell Biol* **23**, 5836–5848 (2003).
63. Li, X. *et al.* Role for KAP1 serine 824 phosphorylation and sumoylation/desumoylation switch in regulating KAP1-mediated transcriptional repression. *J. Biol. Chem.* **282**, 36177–36189 (2007).
64. Bunch, H. & Calderwood, S. K. TRIM28 as a novel transcriptional elongation factor. *BMC Mol. Biol.* **16**, 14 (2015).
65. Czerwińska, P., Mazurek, S. & Wiznerowicz, M. The complexity of TRIM28 contribution to cancer. *J Biomed Sci* **24**, (2017).
66. Bhatia, N. *et al.* MAGE-C2 promotes growth and tumorigenicity of melanoma cells, phosphorylation of KAP1, and DNA damage repair. *J. Invest. Dermatol.* **133**, 759–767 (2013).
67. Chang, C.-W. *et al.* Phosphorylation at Ser473 regulates heterochromatin protein 1 binding and corepressor function of TIF1beta/KAP1. *BMC Molecular Biology* **9**, 61 (2008).
68. Kubota, S. *et al.* Phosphorylation of KRAB-associated protein 1 (KAP1) at Tyr-449, Tyr-458, and Tyr-517 by nuclear tyrosine kinases inhibits the association of KAP1 and heterochromatin protein 1α (HP1α) with heterochromatin. *J. Biol. Chem.* **288**, 17871–17883 (2013).
69. Cheng, C.-T., Kuo, C.-Y. & Ann, D. K. KAPtain in charge of multiple missions: Emerging roles of KAP1. *World J Biol Chem* **5**, 308–320 (2014).
70. Kattapuram, T., Yang, S., Maki, J. L. & Stone, J. R. Protein kinase CK1alpha regulates mRNA binding by heterogeneous nuclear ribonucleoprotein C in response to physiologic levels of hydrogen peroxide. *J. Biol. Chem.* **280**, 15340–15347 (2005).
71. Chang, B. Y., Harte, R. A. & Cartwright, C. A. RACK1: a novel substrate for the Src protein-tyrosine kinase. *Oncogene* **21**, 7619–7629 (2002).

72. Peng, R. *et al.* Forced downregulation of RACK1 inhibits glioma development by suppressing Src/Akt signaling activity. *Oncol. Rep.* **30**, 2195–2202 (2013).
73. Chakrabandhu, K. *et al.* An Evolution-Guided Analysis Reveals a Multi-Signaling Regulation of Fas by Tyrosine Phosphorylation and its Implication in Human Cancers. *PLoS Biol.* **14**, e1002401 (2016).
74. Chakrabandhu, K. & Hueber, A.-O. Fas Versatile Signaling and Beyond: Pivotal Role of Tyrosine Phosphorylation in Context-Dependent Signaling and Diseases. *Front Immunol* **7**, 429 (2016).
75. Chen, J.-T. *et al.* Identification of the lamin A/C phosphoepitope recognized by the antibody P-STM in mitotic HeLa S3 cells. *BMC Biochem.* **14**, 18 (2013).
76. Kochin, V. *et al.* Interphase phosphorylation of lamin A. *J Cell Sci* **127**, 2683–2696 (2014).
77. Berwick, D. C., Hers, I., Heesom, K. J., Moule, S. K. & Tavaré, J. M. The Identification of ATP-citrate Lyase as a Protein Kinase B (Akt) Substrate in Primary Adipocytes. *J. Biol. Chem.* **277**, 33895–33900 (2002).
78. Porstmann, T. *et al.* SREBP Activity Is Regulated by mTORC1 and Contributes to Akt-Dependent Cell Growth. *Cell Metab* **8**, 224–236 (2008).
79. Lewis, C. A., Townsend, P. A. & Isacke, C. M. Ca(2+)/calmodulin-dependent protein kinase mediates the phosphorylation of CD44 required for cell migration on hyaluronan. *Biochem. J.* **357**, 843–850 (2001).
80. Martin, T. A., Harrison, G., Mansel, R. E. & Jiang, W. G. The role of the CD44/ezrin complex in cancer metastasis. *Critical Reviews in Oncology/Hematology* **46**, 165–186 (2003).
81. Thorne, R. F., Legg, J. W. & Isacke, C. M. The role of the CD44 transmembrane and cytoplasmic domains in co-ordinating adhesive and signalling events. *J. Cell. Sci.* **117**, 373–380 (2004).
82. Callera, G. E. *et al.* Regulation of the novel Mg2+ transporter transient receptor potential melastatin 7 (TRPM7) cation channel by bradykinin in vascular smooth muscle cells. *J. Hypertens.* **27**, 155–166 (2009).
83. Dorovkov, M. V., Kostyukova, A. S. & Ryazanov, A. G. Phosphorylation of annexin A1 by TRPM7 kinase: a switch regulating the induction of an α-helix. *Biochemistry* **50**, 2187–2193 (2011).
84. Bailly, E. *et al.* Phosphorylation of two small GTP-binding proteins of the Rab family by p34cdc2. *Nature* **350**, 715–718 (1991).
85. Yang, X.-Z. *et al.* Rab1 in cell signaling, cancer and other diseases. *Oncogene* **35**, 5699–5704 (2016).
86. Li, S. *et al.* Functional link of BRCA1 and ataxia telangiectasia gene product in DNA damage response. *Nature* **406**, 210–215 (2000).
87. You, Z. & Bailis, J. M. DNA damage and decisions: CtIP coordinates DNA repair and cell cycle checkpoints. *Trends in Cell Biology* **20**, 402–409 (2010).
88. Prados-Carvajal, R., López-Saavedra, A., Cepeda-García, C., Jimeno, S. & Huertas, P. Multiple roles of the splicing complex SF3B in DNA end resection and homologous recombination. *DNA Repair* **66–67**, 11–23 (2018).
89. Salvi, M. *et al.* CK2 involvement in ESCRT-III complex phosphorylation. *Arch. Biochem. Biophys.* **545**, 83–91 (2014).
90. Peng, K. *et al.* Stress-induced endocytosis and degradation of epidermal growth factor receptor are two independent processes. *Cancer Cell International* **16**, 25 (2016).
91. Kumar, S. & Hamilton, A. D. α-Helix Mimetics as Modulators of Aβ Self-Assembly. *J. Am. Chem. Soc.* **139**, 5744–5755 (2017).
92. Kumar, S., Henning-Knechtel, A., Magzoub, M. & Hamilton, A. D. Peptidomimetic-Based Multidomain Targeting Offers Critical Evaluation of Aβ Structure and Toxic Function. *J. Am. Chem. Soc.* **140**, 6562–6574 (2018).
