# Supplementary material for: Protein mimetic amyloid inhibitor potently abrogates cancer-associated mutant p53 aggregation and restores tumor suppressor function
Source: Nat Commun. 2021 Jun 25;12:3962. doi: 10.1038/s41467-021-23985-1 (PMC8233319; doi:10.1038/s41467-021-23985-1)
Supplement: Supplementary file 3 — Reporting Summary [file 41467_2021_23985_MOESM3_ESM.pdf]

nature research

Corresponding author(s): Mazin Magzoub, Sunil Kumar and Andrew D. Hamilton

Last updated by author(s): May 14, 2021

Reporting Summary

Nature Research wishes to improve the reproducibility of the work that we publish. This form provides structure for consistency and transparency in reporting. For further information on Nature Research policies, see our Editorial Policies and the Editorial Policy Checklist.

Statistics

For all statistical analyses, confirm that the following items are present in the figure legend, table legend, main text, or Methods section.

n/a	Confirmed	
☐	☒	The exact sample size (n) for each experimental group/condition, given as a discrete number and unit of measurement
☐	☒	A statement on whether measurements were taken from distinct samples or whether the same sample was measured repeatedly
☐	☒	The statistical test(s) used AND whether they are one- or two-sided<br>*Only common tests should be described solely by name; describe more complex techniques in the Methods section.*
☒	☐	A description of all covariates tested
☒	☐	A description of any assumptions or corrections, such as tests of normality and adjustment for multiple comparisons
☐	☒	A full description of the statistical parameters including central tendency (e.g. means) or other basic estimates (e.g. regression coefficient) AND variation (e.g. standard deviation) or associated estimates of uncertainty (e.g. confidence intervals)
☐	☒	For null hypothesis testing, the test statistic (e.g. F, t, r) with confidence intervals, effect sizes, degrees of freedom and P value noted<br>*Give P values as exact values whenever suitable.*
☒	☐	For Bayesian analysis, information on the choice of priors and Markov chain Monte Carlo settings
☒	☐	For hierarchical and complex designs, identification of the appropriate level for tests and full reporting of outcomes
☐	☒	Estimates of effect sizes (e.g. Cohen's d, Pearson's r), indicating how they were calculated

Our web collection on statistics for biologists contains articles on many of the points above.

Software and code

Policy information about availability of computer code

Data collection	BD FACSDiva software (version 8.0), FV10-ASW Viewer software (version 4.2; Olympus), Gen5 (version 2.0; BioTek, Winooski, VT), Image Lab (version 4.1; Bio-Rad), TOPSPIN NMR software (version 4.0.2; Bruker), Velox TEM and STEM software (version 2.9.0; Thermo Fisher Scientific).
Data analysis	Database for Annotation, Visualization Integrated Discovery (DAVID) bioinformatics tool (version 6.8; https://david.ncifcrf.gov/home.jsp), Fiji image processing software (ImageJ2 core with plugins for scientific image analysis; https://imagej.net/Fiji), FlowJo flow cytometry analysis software (version 10.6; FlowJo LLC, Ashland, OR), Gene set enrichment analysis (GSEA) software (version 2.2.3; http://software.broadinstitute.org/gsea/), Ingenuity Pathway Analysis software (IPA) software (QIAGEN Inc., Redwood City, CA; https://digitalinsights.qiagen.com), JMP genomics software (version 9.1; https://www.jmp.com/en_us/software/genomics-data-analysis-software.html), Prism (version 8.4.2; GraphPad Software, Inc., La Jolla, CA, USA), TOPSPIN NMR software (version 4.0.2; Bruker), Velox TEM and STEM software (version 2.9.0; Thermo Fisher Scientific).

For manuscripts utilizing custom algorithms or software that are central to the research but not yet described in published literature, software must be made available to editors and reviewers. We strongly encourage code deposition in a community repository (e.g. GitHub). See the Nature Research guidelines for submitting code & software for further information.

Data

Policy information about availability of data

All manuscripts must include a data availability statement. This statement should provide the following information, where applicable:

- Accession codes, unique identifiers, or web links for publicly available datasets
- A list of figures that have associated raw data
- A description of any restrictions on data availability

Raw data for Figures 1, 3, 4, 6, 7, and Supplementary Figures 1, 7–12, 14, 15, 20 and 21, are provided in the Source Data File. RNA-Seq data has been deposited in

the Gene Expression Omnibus (GEO) repository (accession code: GSE161952; https://www.ncbi.nlm.nih.gov/geo/query/acc.cgi?acc=GSE161952) at the National Center for Biotechnology Information (NCBI). Proteomics data has been deposited in the MassIVE repository (accession code: MSV000086563; https://doi:10.25345/C5X78B) at the ProteomeXchange Consortium. The following publicly available databases were used in the study: IARC TP53 database (https://p53.iarc.fr), Database for Annotation, Visualization Integrated Discovery (DAVID; https://david.ncifcrf.gov/home.jsp), Molecular Signatures Database (MSigDB; http://www.gsea-msigdb.org/gsea/msigdb/index.jsp) and UniProtKB_TrEMBL database (https://www.uniprot.org/uniprot). All the datasets generated and/or analyzed during the current study are also available from the corresponding authors on reasonable request.

Field-specific reporting

Please select the one below that is the best fit for your research. If you are not sure, read the appropriate sections before making your selection.

[x] Life sciences [ ] Behavioural & social sciences [ ] Ecological, evolutionary & environmental sciences

For a reference copy of the document with all sections, see nature.com/documents/nr-reporting-summary-flat.pdf

Life sciences study design

All studies must disclose on these points even when the disclosure is negative.

Sample size: Power calculation was used to select sample sizes from the NYU Abu Dhabi Institutional Animal Care and Use Committee (NYUAD-IACUC) Protocol (Protocol No. 18-0001).

Data exclusions: No data was excluded from the analysis.

Replication: A minimum of three independent experiments were performed for each test. Sample sizes and statistical data are reported in the figure legends.

Randomization: For in vitro studies, samples were randomly allocated into treatment groups. For the tumor inhibition studies, tumor-bearing mice were randomized into the treatment groups.

Blinding: For in vitro studies, investigators were blinded for all parts of the experiments (treatment, data acquisition and data analysis), and a different investigator carried out each part. For in vivo studies, investigators were blinded for data acquisition and data analysis.

Reporting for specific materials, systems and methods

We require information from authors about some types of materials, experimental systems and methods used in many studies. Here, indicate whether each material, system or method listed is relevant to your study. If you are not sure if a list item applies to your research, read the appropriate section before selecting a response.

Materials & experimental systems

n/a	Involved in the study	
[ ]	[x]	Antibodies
[ ]	[x]	Eukaryotic cell lines
[x]	[ ]	Palaeontology and archaeology
[ ]	[x]	Animals and other organisms
[x]	[ ]	Human research participants
[x]	[ ]	Clinical data
[x]	[ ]	Dual use research of concern

Methods

n/a	Involved in the study	
[x]	[ ]	ChIP-seq
[ ]	[x]	Flow cytometry
[x]	[ ]	MRI-based neuroimaging

Antibodies

Antibodies used: Primary antibodies: Anti-amyloid oligomer (A11, AHB0052; Thermo Fisher Scientific), anti-Bax (E63, ab32503; Abcam), anti-Beta-actin (2A3, sc-517582; Santa Cruz), anti-MDM2 (2A10, ab16895; Abcam), anti-Noxa (114C307, sc-56169; Santa Cruz), anti-p21 (EPR3993, ab109199; Abcam), anti-p53 (DO-1, sc-126; Santa Cruz), anti-p53 (DO-7, sc-47698; Santa Cruz), anti-p53 (PAb 240, sc-99; Santa Cruz), anti-p63 (D2K8X, #13109; Cell Signaling Technology), anti-p73 (EPR19884, ab215038; Abcam).

Secondary antibodies: horseradish peroxidase (HRP)-conjugated anti-rabbit IgG (ab6721; Abcam), horseradish peroxidase (HRP)-conjugated anti-mouse IgG (ab205719), Alexa 488-labeled anti-mouse IgG (ab150117; Abcam), Alexa 594-labeled anti-mouse IgG (ab150120; Abcam).

Dilutions for all antibodies are provided in the manuscript.

Validation: The specificity of the antibodies was validated by the manufacturers as noted on their websites (links provided below for each antibody).
Anti-amyloid oligomer (A11, AHB0052; Thermo Fisher Scientific): Human, Mouse, Rat; WB, IHC, ELISA, IP, Dot Blot, ICC/IF (https://

www.thermofisher.com/antibody/product/Oligomer-A11-Antibody-Polyclonal/AHB0052)
Anti-Bax (E63, ab32503; Abcam): Human, Rat; WB, IP, IHC-P, Sandwich ELISA (https://www.abcam.com/bax-antibody-e63-ab32503.html)
Anti-Beta-actin (2A3, sc-517582; Santa Cruz): Human, Mouse, Rat; WB, IP, IF, IHC-P (https://www.scbt.com/p/beta-actin-antibody-2a3)
Anti-MDM2 (2A10, ab16895; Abcam): Human; WB, ICC, IHC-P, Flow Cyt (https://www.abcam.com/mdm2-antibody-2a10-ab16895.html)
Anti-Noxa (114C307, sc-56169; Santa Cruz): Human, Mouse, Rat; WB, IP, IF, IHC-P (https://www.scbt.com/p/noxa-antibody-114c307)
Anti-p21 (EPR3993, ab109199; Abcam): Human, Mouse, Rat; WB (https://www.abcam.com/p21-antibody-epr3993-ab109199.html)
Anti-p53 (DO-1, sc-126; Santa Cruz): Human, Mouse, Rat; WB, IP, IF, IHC-P, Flow Cyt, ChIP (https://www.scbt.com/p/p53-antibody-do-1)
Anti-p53 (DO-7, sc-47698; Santa Cruz): Human; WB, IP, IF, IHC-P, Flow Cyt, ELISA (https://www.scbt.com/p/p53-antibody-do-7)
Anti-p53 (PAb 240, sc-99; Santa Cruz): Human, Mouse, Rat; WB, IP, IF, IHC-P, Flow Cyt, ELISA (https://www.scbt.com/p/p53-antibody-pab-240)
Anti-p63 (D2K8X, #13109; Cell Signaling Technology): Human, Mouse, Rat; WB, IP, IF, F, ChIP (https://www.cellsignal.com/products/primary-antibodies/p63-a-d2k8x-xp-rabbit-mab/13109)
Anti-p73 (EPR19884, ab215038; Abcam): Human; WB, IHC-P, IP, ChIP (https://www.abcam.com/p73-antibody-epr19884-chip-grade-ab215038.html)

Eukaryotic cell lines

Policy information about cell lines

Cell line source(s)

Human bone (U-2 OS [ATCC no. HTB-96] and Saos-2 [HTB-85]), brain (SH-SY5Y [CRL-2266]), breast (HCC70 [CRL-2315], MCF-7 [HTB-22], MDA-MB-175-VII [HTB-25], MDA-MB-231 [HTB-26] and SK-BR-3 [HTB-30]), colon (COLO 320DM [CCL-220], HT-29 [HTB-38], LS123 [CCL-255] and LS 174T [CL-188]), gastric (AGS [CRL-1739]), leukemia (ARH-77 [CRL-1621] and CESS [TIB-190]), lung (A549 [CCL-185], NCI-H1770 [CRL-5893], NCI-H1882 [CRL-5903], NCI-H2342 [CRL-5941] and NCI-H748 [CRL-5841]), ovarian (OVCAR-3 [HTB-161] and SKOV-3 [HTB-77]), pancreatic (MIA PaCa-2 [CRL-1420]) and renal (CAKI-1 [HTB-46]), cancer cells were all from American Type Culture Collection (ATCC).

Authentication

All cell lines were authenticated by either ATCC or Charles River Laboratories using short tandem repeat (STR) analysis.

Mycoplasma contamination

Testing for mycoplasma contamination was done by Charles River Laboratories, and all cell lines tested negative for mycoplasma contamination.

Commonly misidentified lines
(See ICLAC register)

No commonly misidentified cell lines were used in this study.

Animals and other organisms

Policy information about studies involving animals; ARRIVE guidelines recommended for reporting animal research

Laboratory animals

Athymic nude NU/J mice (Foxn1nu; The Jackson Laboratory, Bar Harbor, ME), female, 6-8 weeks old. Mice were maintained in air-filtered cages with controlled temperature (20 °C) and humidity (50%), in a 12 h light/dark cycle, and fed standard mouse chow (Research Diets; New Brunswick, NJ).

Wild animals

No wild animals were used in this study.

Field-collected samples

No field-collected samples were used in this study.

Ethics oversight

All animal experiments were approved by the NYU Abu Dhabi Institutional Animal Care and Use Committee (NYUAD-IACUC; Protocol No. 18-0001), and were carried out in accordance with the Guide for Care and Use of Laboratory Animals.

Note that full information on the approval of the study protocol must also be provided in the manuscript.

Flow Cytometry

Plots

Confirm that:

- [x] The axis labels state the marker and fluorochrome used (e.g. CD4-FITC).
- [x] The axis scales are clearly visible. Include numbers along axes only for bottom left plot of group (a 'group' is an analysis of identical markers).
- [x] All plots are contour plots with outliers or pseudocolor plots.
- [x] A numerical value for number of cells or percentage (with statistics) is provided.

Methodology

Sample preparation

For the Dead Cell Apoptosis assay, MIA PaCa-2 cells (ATCC no. CRL-1420) were treated with 5 µM ADH-1, ADH-6 or ReACp53 for 24 h at 37 °C. Subsequently, the cells were washed with ice-cold PBS, harvested by trypsinization, centrifuged and re-suspended in 1×annexin-binding buffer (10 mM HEPES, 140 mM NaCl, 2.5 mM CaCl2, pH 7.4) to a density of ~1×10^6 cells/

mL. The cells were then stained with 5 µL Alexa 488-conjugated annexin V and 0.1 µg PI per 100 µL of cell suspension for 15 min at room temperature. Immediately afterwards, fluorescence was measured using flow cytometry (10,000 cells/sample) on a BD FACSAria III cell sorter (BD Biosciences, San Jose, CA).

For the cell cycle analysis, MIA PaCa-2 cells were seeded at a density of 1×10^6 cells/well in 1 mL complete DMEM. After culturing for 24 h, the medium was replaced with serum-free DMEM containing 5 µM ADH-1, ReACp53 or ADH-6, and the cells were incubated for 6 h. Subsequently, the cells were washed with PBS, harvested by trypsinization and centrifuged (800×g for 5 min). The supernatant was discarded, and the cells were washed with PBS and fixed in 70% cold ethanol overnight at 4°C. The cells were then washed with PBS, filtered through a nylon sieve, and centrifuged again (800×g for 5 min), and the supernatant was discarded. The cells were stained with Nuclear Green CCS1 (Abcam) for 30 min at 37 °C and data (10,000 cells/sample) was collected on a BD FACSAria III cell sorter.

Instrument	BD FACSAria III cell sorter (BD Biosciences, San Jose, CA).
Software	Data analysis was performed using FlowJo (version 10.6; FlowJo LLC, Ashland, OR).
Cell population abundance	Samples were filtered prior to experiments using standard operating procedures. For each experiment, at least 10,000 cells/sample (3-4 independent samples) were sorted. Post-sort populations were quantified to determine measured parameters (e.g. apoptosis, cell cycle distribution).
Gating strategy	At least 10,000 cells/sample (3-4 biological replicates), gated to remove cell debris by forward/side scatter.

☒ Tick this box to confirm that a figure exemplifying the gating strategy is provided in the Supplementary Information.
